# Joint blood pressure and resting heart rate trajectories and net adverse clinical events risk following PCI: an inverse probability of treatment weighting analysis

**DOI:** 10.3389/fmed.2026.1914874

**Published:** 2026-09-07

**Authors:** Xinyu Xu, Yingbu Niu, Xueyun Zhao, Shikang Liu, Yuanyuan Shi, Qisheng Yan, Yang Wang, Juanli Li

**Affiliations:** 1 Department of Cardiovascular Medicine, the First Affiliated Hospital of Xi'an Jiaotong University, Xi'an, Shaanxi, China; 2 School of Public Health, Xi’an Jiaotong University Health Science Center, Xi’an, China; 3 Emergency Room of Internal Medicine, Shaanxi Provincial People's Hospital, Xi'an, China; 4 School of Basic Medical Sciences, Health Science Center, Xi'an Jiaotong University, Xi'an, China

**Keywords:** blood pressure, inverse probability, percutaneous coronary intervention, prognosis, trajectory

## Abstract

**Background:**

Evidence is limited on the prospective prognostic value of longitudinal blood pressure (BP) and resting heart rate (RHR) dynamics after percutaneous coronary intervention (PCI).

**Aim:**

This study aimed to identify joint BP-RHR trajectories and evaluate their impact on net adverse clinical events (NACE).

**Methods:**

In a prospective post-PCI cohort, latent class trajectory modeling identified 6-month patterns of systolic BP, diastolic BP, and RHR, classifying patients into four joint trajectories. Inverse probability of treatment weighting (IPTW) was employed to rigorously balance baseline confounders. The primary endpoint was NACE. The incremental predictive value of the joint trajectories was assessed using the C-index, continuous net reclassification improvement (cNRI), and integrated discrimination improvement (IDI).

**Results:**

Among 736 patients (71 NACE events during follow-up), four distinct joint trajectory groups were identified: Double Stable (*n* = 329), BP-Fluctuating (*n* = 215), RHR-Fluctuating (*n* = 108), and Double Fluctuating (*n* = 84). After IPTW adjustment, compared with the Double Stable group, the risk of NACE was significantly elevated in the BP-Fluctuating group (HR: 2.21, 95% CI: 1.23–3.97), the RHR-Fluctuating group (HR: 1.85, 95% CI: 1.09–3.13), and peaked in the Double Fluctuating group (HR: 2.69, 95% CI: 1.18–6.14). Furthermore, incorporating the joint trajectory groups into a baseline clinical risk model significantly improved prognostic discrimination and reclassification, with significant increases in the C-index (*p* < 0.001), IDI improvement (*p* = 0.033), and continuous net reclassification improvement.

**Conclusion:**

Joint BP and RHR trajectories effectively stratify post-PCI NACE risk. Patients with combined fluctuations face the highest risk, highlighting the need for dynamic hemodynamic monitoring over static single-point assessments.

## Introduction

Acute myocardial infarction (AMI) is a leading cause of cardiovascular morbidity and mortality worldwide, frequently resulting in irreversible myocardial damage, life-threatening arrhythmias, and sudden cardiac death if left untreated (1, 2). Early restoration of coronary perfusion is an important component of AMI management (3). Percutaneous coronary intervention (PCI) is a critical catheter-based revascularization procedure that utilizes balloon angioplasty and stent implantation to mechanically dilate obstructed coronary arteries and restore essential blood flow to the ischemic myocardium (3, 4). Over the past decades, PCI has substantially changed coronary artery disease management and has been associated with improved survival (5–7). However, despite high procedural success rates and advances in stent technologies, post-PCI patients remain at a substantially high risk for recurrent adverse outcomes (8, 9). Large-scale real-world registries and clinical trials have demonstrated that the incidence of net adverse clinical events (NACE) remains a clinical concern, affecting approximately 15% of patients within the first year and increasing to nearly 24 to 37% at three years following the index procedure (10, 11). This vital residual risk during the secondary prevention phase underscores the critical need for optimal post-discharge management and precise risk stratification to identify vulnerable patients who are prone to poor outcomes.

Hemodynamic parameters, specifically blood pressure (BP) and resting heart rate (RHR), are fundamental modifiable therapeutic targets in the post-PCI setting (3, 12). Large-scale epidemiological cohort studies have consistently demonstrated that in the post-PCI population, elevated BP or high resting RHR is independently associated with an increased risk of recurrent ischemic and net adverse clinical events (13–15). For instance, a recent large-scale cohort study of patients undergoing PCI demonstrated that uncontrolled blood pressure was associated with an increased risk of adverse cardiovascular outcomes (16). Similarly, another prospective study revealed that an elevated RHR after PCI was independently associated with a significantly higher risk of subsequent major adverse clinical events (17). Nevertheless, current clinical guidelines and prognostic models predominantly rely on single, static baseline measurements. However, these cross-sectional baseline data are often confounded by the masking effect of intensive acute medical interventions (e.g., the administration of high-dose *β*-blockers and vasodilators) during the index hospitalization (18). Because such snapshot assessments fail to reflect the patient’s true physiological phenotype and medication adherence, it is imperative to capture the continuous hemodynamic variations of patients over time through dynamic, longitudinal tracking (19).

Recently, latent class trajectory modeling (LCTM) has emerged as a robust analytical approach to capture the cumulative burden of longitudinal clinical data (20, 21). While several studies have demonstrated the prognostic utility of isolated BP or RHR trajectories for predicting adverse outcomes in other cardiovascular diseases, they predominantly evaluate these parameters independently (22, 23). To our knowledge, no previous study has investigated the prognostic impact of joint longitudinal BP and RHR trajectories specifically in the post-PCI population. Human hemodynamics, however, operate as an integrated system (24). The physiological interplay between BP and RHR, such as synergistic escalation driven by sympathetic overactivation or progressive ventricular remodeling, cannot be fully captured by evaluating either parameter in isolation. Therefore, integrating both BP and RHR into a joint analytical framework can more comprehensively elucidate their complex physiological interplay. This integrated approach provides a more accurate characterization of the patient’s actual hemodynamic profile.

Therefore, the present study aimed to: (1) identify distinct joint longitudinal trajectories of BP and RHR during the crucial 6-month follow-up window in a cohort of post-PCI patients; (2) evaluate the association between these joint dynamic trajectories and the subsequent risk of NACE; and (3) determine whether this longitudinal tracking strategy provides incremental predictive value over traditional risk factors and conventional single baseline measurements.

## Methods

### Study population

This prospective observational cohort study was conducted at the First Affiliated Hospital of Xi’an Jiaotong University, a large tertiary medical center in Northwest China. Between March 2024 and May 2025, we prospectively enrolled consecutive adult patients who were admitted and scheduled to undergo PCI. To construct latent class trajectories and monitor outcomes, all enrolled participants were prospectively scheduled for standardized clinical follow-up visits at 1 month, 3 months, and 6 months post-PCI, followed by continuous surveillance for incident adverse clinical events.

The initial study population encompassed a broad spectrum of coronary artery disease. Following the 6-month landmark period and the application of pre-defined criteria, the final analytical cohort comprised 736 eligible patients. Based on their initial clinical presentation, these patients were broadly categorized into two primary groups: 565 individuals with acute coronary syndrome (ACS) and 171 individuals with chronic coronary syndrome (CCS).

To ensure the rigorousness of this prospective cohort, consecutive adult patients scheduled for PCI were strictly screened for eligibility. Initially, patients were excluded prior to enrollment if they met the following criteria: (1) history of previous coronary artery bypass grafting, or (2) presence of severe concomitant diseases with a life expectancy of less than 1 year. After applying these initial screening criteria, a total of 986 eligible patients were successfully enrolled, provided written informed consent, and completed the initial PCI procedure with stent implantation.

**Figure 1:**
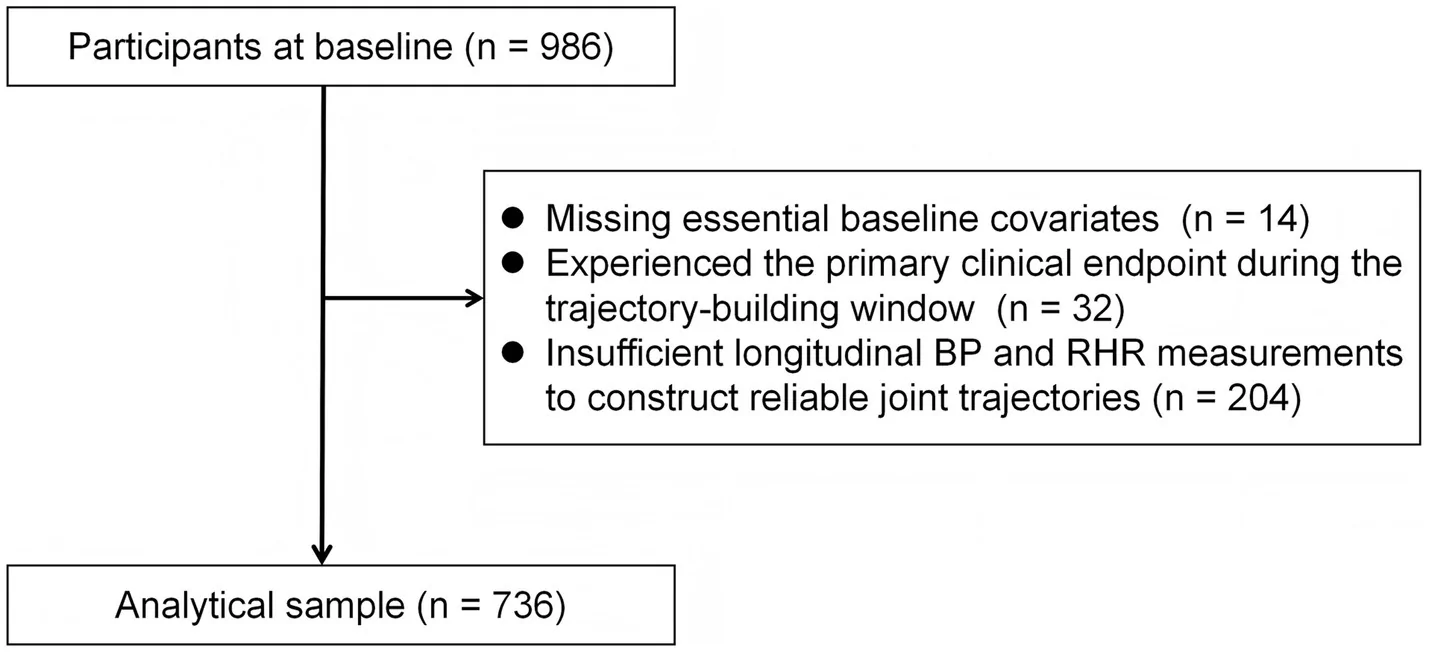
Participant flowchart.

During the prospective follow-up and the 6-month trajectory-building phase, patients were further excluded from the final analysis if they: (1) had missing essential baseline clinical or angiographic covariates (*n* = 14); (2) experienced the primary clinical endpoint or died during the initial 6-month window, thereby violating the immortal time assumption (*n* = 32); or (3) failed to complete the requisite longitudinal blood pressure and RHR measurements, or were lost to follow-up (*n* = 204). Consequently, the final analytical cohort comprised 736 patients (Figure 1). Among these 204 lost-to-follow-up patients, the primary reasons for attrition were transfer of care to other hospitals (*n* = 109, 53.4%), invalid contact information (*n* = 57, 27.9%), and voluntary withdrawal from the study (*n* = 38, 18.6%).

To reduce immortal time bias and enable trajectory classification for blood pressure and RHR, we applied a strict 6-month landmark analysis. Consequently, the “Time Zero” for the survival analysis was designated as exactly 6 months post-PCI. For the eligible analytical cohort, the prognostic follow-up time was calculated continuously from this 6-month landmark point until the occurrence of the primary clinical endpoint, death from any cause, or administrative censoring. To standardize the observation window, administrative censoring was meticulously defined as the date of the last available clinical contact or the definitive database lock date (February 22, 2026), whichever occurred first.

The study protocols rigorously complied with the ethical guidelines of the Declaration of Helsinki and were approved by the Institutional Review Board of the hospital (Approval No.: XJTU1AF2024LSYY-192). All enrolled participants provided written informed consent before study initiation. The reporting of this manuscript adhered to the Strengthening the Reporting of Observational Studies in Epidemiology guidelines (25).

### Hemodynamic measurement and exposure definition

To ensure the rigorousness of the longitudinal data and minimize transient physiological fluctuations, all BP and RHR measurements were strictly obtained by trained healthcare professionals according to standardized clinical guidelines. Specifically, measurements were performed after patients had rested quietly in a seated position for at least 5 min, and patients were required to avoid caffeine, smoking, and strenuous exercise for at least 30 min prior to the assessment to prevent acute sympathetic interference. During each visit, three consecutive measurements of systolic BP (SBP), diastolic BP (DBP), and RHR were recorded at 1- to 2-min intervals using validated automated oscillometric sphygmomanometers. The average of these three readings was calculated and documented as the representative hemodynamic value for that specific visit.

For trajectory construction, these standardized hemodynamic parameters were systematically collected at four pre-defined time points during the trajectory-building window: at discharge (post-PCI baseline), and at 1-month, 3-month, and 6-month outpatient follow-up visits. In our analytical framework, we adopted a progressive modeling strategy. Initially, LCTM was applied independently to the longitudinal data of SBP, DBP, and RHR. This foundational step allowed us to identify distinct, parameter-specific temporal patterns and evaluate their individual prognostic implications. Building upon these isolated trajectories, and recognizing the integrated physiological interplay between blood pressure and RHR, we further advanced our analysis by constructing a joint trajectory model. By simultaneously incorporating the repeated measures of SBP, DBP, and RHR across the 6-month window into a unified LCTM framework, we identified mutually exclusive, synergistic hemodynamic phenotypes. Ultimately, while the isolated trajectories were analyzed to establish foundational associations, the specific joint BP-RHR trajectory group to which a patient was assigned served as the primary exposure variable in this study.

We selected the 6-month post-PCI time point as the landmark for two primary reasons: (1) this window represents the critical early secondary prevention phase after PCI, during which hemodynamic parameters stabilize and guideline-directed medication titration is typically completed; (2) the landmark approach strictly eliminates immortal time bias, as only patients who survived and remained event-free during the trajectory-building period were included in the prognostic analysis.

### Ascertainment of NACE and follow-up

The primary endpoint ascertained during this follow-up period was the occurrence of NACE. Recognizing the delicate competing risks between ischemic and hemorrhagic complications in the post-PCI setting, NACE was meticulously defined as a composite of major adverse cardiovascular events and clinically significant major bleeding. Specifically, the ischemic components of NACE included all-cause mortality, non-fatal myocardial infarction, ischemic stroke, and ischemia-driven target lesion revascularization (26). The hemorrhagic component was strictly defined as major bleeding corresponding to types 3, 4, or 5 according to the Bleeding Academic Research Consortium criteria (27). To improve consistency of endpoint adjudication, all suspected clinical events were systematically reviewed and adjudicated by an independent clinical event committee (CEC). The CEC comprised two experienced cardiologists who were completely blinded to the patients’ baseline characteristics and their assigned hemodynamic trajectory classifications. In cases of disagreement, consensus was reached through consultation with a third senior cardiologist. Longitudinal follow-up data were comprehensively ascertained through integrated electronic medical records, routine outpatient clinic visits, and structured telephone interviews conducted by trained clinical research coordinators. Patients were continuously followed until the occurrence of the primary endpoint, death, or the uniform administrative censoring date (February 22, 2026), whichever occurred first. Complete follow-up was achieved in 98.2% of the eligible analytical cohort after the 6-month landmark period, with only 13 patients lost to surveillance.

### Assessment of other covariates

Baseline data were systematically extracted from the hospital’s electronic medical records (EMR) system by trained clinical researchers. Demographic characteristics, including age and sex, as well as lifestyle factors, specifically smoking and alcohol drinking status (both categorized as never, current, or former), were obtained through standardized admission interviews. Trained nursing staff measured body height and weight upon admission, and body mass index (BMI) was computed as weight in kilograms divided by height in meters squared.

Detailed personal medical history, encompassing diabetes mellitus (DM), hyperlipidemia, hyperuricemia, stroke, heart failure (HF), anemia, and arrhythmia, was identified based on prior physician diagnoses documented in the medical records. The severity of heart failure was further stratified using the New York Heart Association (NYHA) functional classification (ranging from Normal/Class 0 to Class IV) (28). Given the specific post-PCI nature of this cohort, comprehensive angiographic and interventional characteristics were retrieved from cardiac catheterization laboratory reports. The extent of coronary artery disease was assessed by identifying the presence of lesions in the left main (LM) coronary artery, left anterior descending (LAD) artery, left circumflex (LCX) artery, and right coronary artery (RCA), and was further categorized by the number of diseased vessels (multivessel disease: 0 to 3 vessels). Procedural details, including the specific deployment locations of stents (LM, LAD, LCX, and RCA) and the total number of stents implanted, were precisely recorded.

Regarding other comorbidities that may potentially influence hemodynamic profiles, such as thyroid dysfunction and hematological malignancies, these conditions were not formally included as independent covariates in the primary adjustment set. The primary rationale for this exclusion was the very low prevalence of these disorders in our cohort (<2% for thyroid disease and <1% for hematological diseases). Instead, we included complete blood count parameters and serum creatinine as surrogate markers of systemic hematological and renal status to partially account for relevant pathophysiological effects.

Baseline laboratory parameters were derived from fasting venous blood samples typically collected within 24 h of admission and analyzed at the hospital’s central laboratory. These included complete blood count (white blood cell [WBC], hemoglobin [Hb], and platelets [PLT]), glucose metabolism indicators (fasting blood glucose [FBG] and glycated hemoglobin [HbA1c]), renal function (creatinine [Cr] and serum potassium [K]), and lipid profiles (high-density lipoprotein cholesterol [HDL-C], low-density lipoprotein cholesterol [LDL-C], and triglycerides [TG]). Baseline cardiac function, specifically the left ventricular ejection fraction (LVEF), was evaluated using standard transthoracic echocardiography (29). Finally, discharge medications were meticulously recorded to reflect baseline pharmacological treatments, including the prescription of *β*-blockers, angiotensin-converting enzyme inhibitors or angiotensin II receptor blockers (ACEI/ARB), statins, P2Y12 inhibitors, and aspirin (30).

### Statistical analyses

All statistical analyses were performed using R version 4.2.3 (R Foundation for Statistical Computing, Vienna, Austria), utilizing packages including Weight, survey, and cobalt. A two-sided *p* < 0.05 was considered statistically significant.

The normality of continuous variables was comprehensively evaluated using the Shapiro–Wilk test supplemented by histogram and quantile-quantile (Q-Q) plot visualization, given that the Kolmogorov–Smirnov test is overly sensitive to minor deviations from normality in large sample sizes; normally distributed continuous variables were expressed as means and standard deviations (SDs), whereas skewed variables were reported as medians and interquartile ranges (IQRs). Categorical variables were presented as frequencies and percentages. Baseline characteristics of the unweighted cohort across the different hemodynamic trajectory groups were compared using one-way analysis of variance or the Kruskal-Wallis test for continuous variables, and the Chi-square test or Fisher’s exact test for categorical variables, as appropriate.

To identify the longitudinal patterns of BP and RHR over the initial 6-month landmark period, LCTM was performed. We iteratively evaluated models ranging from 1 to 6 classes for systolic BP, diastolic BP, and RHR. The optimal number of classes for each parameter was determined based on a comprehensive assessment of model fit indices (e.g., lowest Bayesian Information Criterion (BIC) and Akaike Information Criterion (AIC), adequate entropy, and clinically viable minimum class size). Subsequently, a joint LCTM framework was utilized to categorize patients into mutually exclusive joint BP-RHR phenotypes. Exhaustive technical details regarding the LCTM construction—including the handling of missing data, specification of trajectory function forms, average posterior probabilities, and complete fit indices are comprehensively described in the Supplementary material (Methods) and Supplementary Tables 1,2.

To rigorously account for baseline confounding and to overcome the potential limitation of a low events-per-variable (EPV) ratio in traditional multivariable modeling, we implemented an Inverse Probability of Treatment Weighting (IPTW) approach using generalized propensity scores. First, a multinomial logistic regression model was utilized to estimate the probability of each patient being classified into one of the four joint trajectory groups. This model systematically incorporated 17 clinically relevant baseline covariates: age, sex, body mass index, smoking status, clinical presentation (acute versus chronic coronary syndrome), diabetes mellitus, heart failure, stroke, number of stents in the LM coronary artery, total number of stents, use of *β*-blockers, use of ACEI/ARB, WBC count, platelet count, creatinine, LDL-C, and triglycerides. Patients lost to follow-up before the administrative censoring date, and those who remained event-free at the database lock date, were handled as right-censored observations in all survival analyses.

To reduce the variance and potential instability caused by extreme weights, the calculated weights were symmetrically truncated at the 1st and 99th percentiles. The balance of covariates across the trajectory groups before and after weighting was assessed using standardized mean differences (SMDs), with an SMD < 0.1 was considered to indicate good balance, and an SMD < 0.2 was considered clinically acceptable. After IPTW weighting, all 17 covariates achieved optimal balance (all SMDs < 0.10); thus, no additional covariates were included in the primary weighted Cox proportional hazards models. Finally, the IPTW-adjusted hazard ratios (HRs) and 95% confidence intervals (CIs) for incident NACE were estimated. In all weighted Cox models, robust standard errors based on the sandwich variance estimator were applied to appropriately account for the intra-individual correlation and pseudo-population clustering introduced by the weighting procedure.

Cumulative event rates for incident NACE were estimated using weighted Kaplan–Meier curves, and differences in NACE-free survival among the joint trajectory groups were compared using a weighted log-rank test. Subsequently, a weighted Cox proportional hazards model, incorporating the trajectory group variable (and any unbalanced covariates, if applicable), was conducted on the pseudo-population to estimate the independent hazard ratios (HRs) and 95% confidence intervals (CIs) for the risk of NACE. Robust standard errors were utilized in the weighted Cox models to account for the artificial inflation of the sample size.

Restricted cubic spline (RCS) models with 3 knots placed at the 10th, 50th, and 90th percentiles of the baseline hemodynamic parameters were used to evaluate the non-linear association between single baseline measurements and NACE risk.

Exploratory subgroup analyses were conducted to evaluate the consistency of the prognostic impact of the joint trajectories across various pre-specified clinical strata, including age, sex, smoking status, presence of diabetes, and heart failure. Interaction effects between the trajectory groups and these stratification variables were examined using likelihood ratio tests. Furthermore, to quantify the incremental predictive value of incorporating the joint BP-RHR trajectory groups into a baseline clinical risk model, we calculated the change in Harrell’s concordance index (C-index), the integrated discrimination improvement (IDI), and the continuous net reclassification improvement (NRI) within the original cohort. The 95% CIs and *p* values for IDI and cNRI were generated using bootstrap resampling with 1,000 iterations to correct for overfitting bias in the single-center cohort. The statistical significance of the C-index improvement was tested using DeLong’s method.

## Results

### Identification of longitudinal trajectories

**Figure 2:**
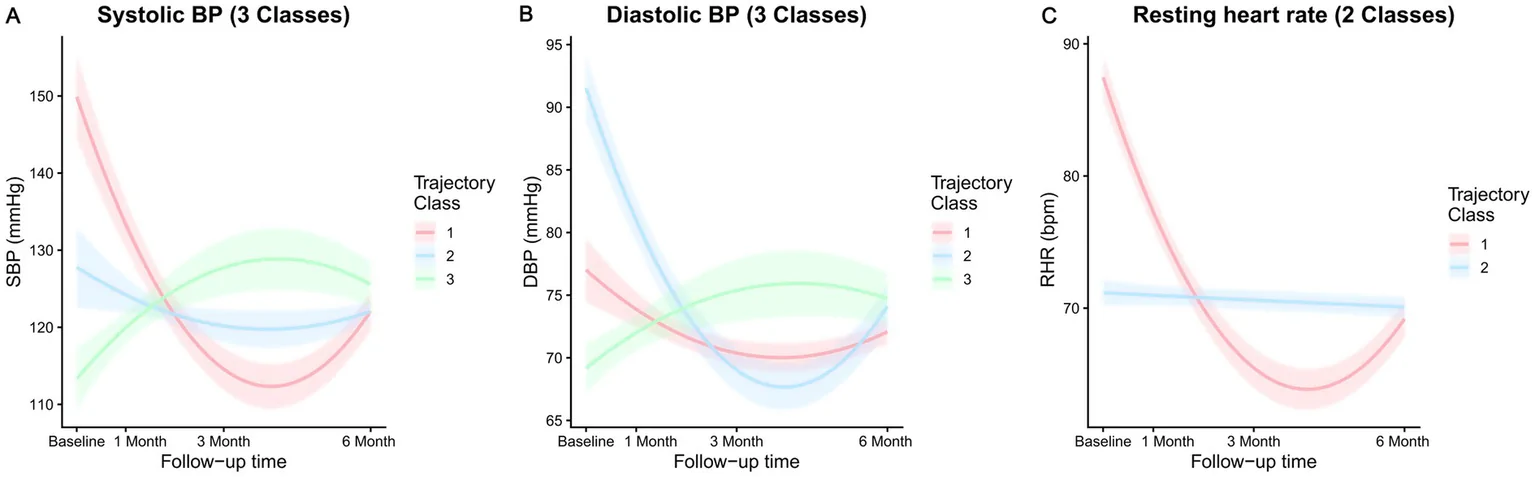
Distinct longitudinal trajectory classes of individual hemodynamic parameters over the 6-month follow-up period. **(A)** Systolic blood pressure (SBP), **(B)** diastolic blood pressure (DBP), **(C)** resting heart rate (RHR). The solid lines represent the estimated mean values for each latent trajectory class, and the shaded bands indicate the corresponding 95% confidence intervals. The x-axis reflects the follow-up time from baseline to 6 months.

Longitudinally, the optimal latent trajectory classes were identified as 3 for SBP, 3 for DBP, and 2 for RHR (Figures 2A–C; Supplementary Tables 1,2), with acceptable model fit in Supplementary Figures 1–3. SBP and DBP trajectories each exhibited 3 corresponding temporal patterns (stable, high-decreasing-increasing, low-increasing), with distinct reference assignments: Group 2 (stable pattern) for SBP, and Group 1 (stable pattern) for DBP. RHR trajectories were categorized into 2 groups: Group 2 (stable, reference) and Group 1 (fluctuating). Ultimately, integrating these isolated parameters classified patients into 4 distinct joint BP-RHR trajectory groups (Supplementary Tables 3–5).

### Baseline characteristics of study participants

There were no significant differences in baseline demographic and clinical characteristics between the included and excluded patients (all *p* > 0.05, Supplementary Table 6). The baseline blood pressure and RHR control status of the study participants is summarized in Table 1 and Supplementary Table 7. Based on this initial status, the cohort was stratified into four distinct joint groups: Group 1 (Double Stable, *n* = 329), Group 2 (BP-Fluctuating, *n* = 215), Group 3 (RHR-Fluctuating, *n* = 108), and Group 4 (Double Fluctuating, *n* = 84). During a median prognostic follow-up of 237 days (interquartile range: 156–292 days) post-landmark, a total of 71 primary clinical endpoint events occurred among the 736 eligible patients. Baseline clinical characteristics were generally well-balanced across the groups. However, prior to IPTW weighting, patients with uncontrolled RHR exhibited a significantly higher prevalence of heart failure and greater utilization of *β*-blockers (both *p* < 0.001). Additional statistically significant differences were observed in clinical presentations (ACS vs. CCS, *p* = 0.002), stents deployed in the left main artery (*p* = 0.007), as well as baseline levels of white blood cells, platelets, and triglycerides (all *p* < 0.05). Longitudinally, the Double Stable group maintained optimal hemodynamic levels over the 6-month period, whereas the “Uncontrolled” groups presented with pronounced baseline elevations that experienced substantial treatment-related declines, yet their overall trajectories remained clinically distinct (Supplementary Table 8). After IPTW weighting, all 17 covariates achieved excellent balance, with all standardized mean differences (SMDs) below 0.11 (maximum SMD = 0.105 for body mass index), indicating negligible residual confounding between groups.

**Table 1:** Baseline characteristics of study participants stratified by BP and RHR control status.

Characteristics	Double stable (*n* = 329)	BP-fluctuating (*n* = 215)	RHR-fluctuating (*n* = 108)	Double fluctuating (*n* = 84)	*p* value
Demographics & lifestyle					
Age, years (mean ± SD)	61.36 ± 10.26	60.71 ± 9.81	60.95 ± 10.43	59.89 ± 11.48	0.674
Sex, *n* (%)					0.190
Male	257 (78.1)	172 (80.0)	77 (71.3)	60 (71.4)	
Female	72 (21.9)	43 (20.0)	31 (28.7)	24 (28.6)	
BMI, kg/m^2^ (mean ± SD)	25.05 ± 3.07	24.77 ± 2.88	24.54 ± 3.19	24.27 ± 3.40	0.144
Smoking, *n* (%)					0.827
Never	191 (58.1)	117 (54.4)	61 (56.5)	49 (58.3)	
Current	109 (33.1)	78 (36.3)	33 (30.6)	26 (31.0)	
Former	29 (8.8)	20 (9.3)	14 (13.0)	9 (10.7)	
Drinking, *n* (%)					0.868
Never	265 (80.5)	171 (79.5)	84 (77.8)	70 (83.3)	
Current	61 (18.5)	42 (19.5)	24 (22.2)	13 (15.5)	
Former	3 (0.9)	2 (0.9)	0 (0.0)	1 (1.2)	
Clinical presentation & history					
Clinical presentation, *n* (%)					0.002
ACS	257 (78.1)	175 (81.4)	82 (75.9)	51 (60.7)	
CCS	72 (21.9)	40 (18.6)	26 (24.1)	33 (39.3)	
Diabetes mellitus, *n* (%)	117 (35.6)	68 (31.6)	33 (30.6)	29 (34.5)	0.700
Hyperlipidemia, *n* (%)	65 (19.8)	43 (20.0)	24 (22.2)	14 (16.7)	0.820
Hyperuricemia, *n* (%)	6 (1.8)	3 (1.4)	3 (2.8)	1 (1.2)	0.808
Stroke, *n* (%)	52 (15.8)	28 (13.0)	14 (13.0)	16 (19.0)	0.520
Heart failure, *n* (%)	15 (4.6)	17 (7.9)	16 (14.8)	14 (16.7)	<0.001
NYHA functional class, *n* (%)					0.155
Normal/0	79 (24.0)	45 (20.9)	25 (23.1)	21 (25.0)	
Class I	33 (10.0)	24 (11.2)	16 (14.8)	11 (13.1)	
Class II	202 (61.4)	127 (59.1)	60 (55.6)	40 (47.6)	
Class III	14 (4.3)	17 (7.9)	5 (4.6)	10 (11.9)	
Class IV	1 (0.3)	2 (0.9)	2 (1.9)	2 (2.4)	
Anemia, *n* (%)	5 (1.5)	3 (1.4)	3 (2.8)	3 (3.6)	0.522
Arrhythmia, *n* (%)	33 (10.0)	26 (12.1)	14 (13.0)	11 (13.1)	0.750
Angiography & interventions					
Left main disease, *n* (%)	42 (12.8)	28 (13.0)	15 (13.9)	15 (17.9)	0.668
LAD lesion, *n* (%)	284 (86.3)	175 (81.4)	94 (87.0)	78 (92.9)	0.071
LCX lesion, *n* (%)	234 (71.1)	154 (71.6)	73 (67.6)	66 (78.6)	0.408
RCA lesion, *n* (%)	218 (66.3)	150 (69.8)	81 (75.0)	54 (64.3)	0.294
Multivessel disease, *n* (%)					0.235
0-vessel	11 (3.3)	16 (7.4)	3 (2.8)	3 (3.6)	
1-vessel	59 (17.9)	29 (13.5)	21 (19.4)	12 (14.3)	
2-vessel	100 (30.4)	60 (27.9)	25 (23.1)	21 (25.0)	
3-vessel	159 (48.3)	110 (51.2)	59 (54.6)	48 (57.1)	
Stents in LM, mean ± SD	0.13 ± 0.37	0.07 ± 0.26	0.08 ± 0.31	0.23 ± 0.52	0.007
Stents in LAD, mean ± SD	0.80 ± 0.95	0.91 ± 0.98	0.94 ± 1.03	0.77 ± 0.87	0.342
Stents in LCX, mean ± SD	0.25 ± 0.57	0.23 ± 0.54	0.28 ± 0.59	0.25 ± 0.53	0.926
Stents in RCA, mean ± SD	0.46 ± 0.83	0.49 ± 0.89	0.46 ± 0.85	0.45 ± 0.91	0.979
Total number of stents, mean ± SD	1.63 ± 1.05	1.71 ± 1.00	1.77 ± 1.04	1.70 ± 0.92	0.630
Discharge medications					
β-blockers, *n* (%)	211 (64.1)	142 (66.0)	91 (84.3)	67 (79.8)	<0.001
ACEI/ARB, *n* (%)	63 (19.1)	42 (19.5)	16 (14.8)	12 (14.3)	0.541
Statin, *n* (%)	313 (95.1)	204 (94.9)	103 (95.4)	77 (91.7)	0.620
P2Y12 inhibitors, *n* (%)	327 (99.4)	211 (98.1)	107 (99.1)	82 (97.6)	0.429
Aspirin, *n* (%)	303 (92.1)	200 (93.0)	103 (95.4)	77 (91.7)	0.685
Laboratory results & echo					
WBC, 10^9^/L (median [IQR])	6.91 [5.19, 8.32]	7.03 [5.50, 8.86]	7.08 [5.40, 9.17]	7.53 [6.39, 9.89]	0.006
Hemoglobin, g/L (mean ± SD)	138.90 ± 17.15	136.91 ± 21.52	139.96 ± 21.11	136.44 ± 25.88	0.435
Platelets, 10^9^/L (mean ± SD)	201.39 ± 59.27	202.96 ± 61.24	218.71 ± 72.11	220.36 ± 69.75	0.013
HbA1c, % (mean ± SD)	6.69 ± 1.42	6.52 ± 1.41	6.60 ± 1.48	6.58 ± 1.35	0.582
FBG, mmol/L (mean ± SD)	6.40 ± 2.50	6.14 ± 2.26	6.16 ± 1.80	6.53 ± 2.69	0.428
Potassium, mmol/L (mean ± SD)	3.96 ± 0.37	3.96 ± 0.40	3.94 ± 0.37	4.06 ± 0.36	0.132
Creatinine, μmol/L (median [IQR])	72.0 [60.0, 100.0]	74.0 [61.0, 107.0]	80.0 [59.8, 116.0]	75.0 [63.0, 126.0]	0.407
HDL-C, mmol/L (mean ± SD)	1.05 ± 0.39	1.04 ± 0.37	1.11 ± 0.38	1.07 ± 0.34	0.408
LDL-C, mmol/L (mean ± SD)	2.32 ± 1.21	2.29 ± 1.30	2.43 ± 1.07	2.39 ± 0.98	0.741
Triglycerides, mmol/L (median [IQR])	3.75 [2.94, 4.58]	3.53 [2.83, 4.46]	3.88 [3.24, 4.68]	4.08 [3.33, 4.75]	0.015
LVEF, % (mean ± SD)	58.22 ± 11.94	58.31 ± 11.75	57.28 ± 13.73	57.03 ± 12.32	0.766

^IPTW, inverse probability of treatment weighting; SMD, standardized mean difference; BMI, body mass index; ACS, acute coronary syndrome; CCS, chronic coronary syndrome; NYHA, New York Heart Association; LM, left main; LAD, left anterior descending; LCX, left circumflex; RCA, right coronary artery; ACEI, angiotensin-converting enzyme inhibitor; ARB, angiotensin receptor blocker; WBC, white blood cell; HbA1c, glycated hemoglobin; FBG, fasting blood glucose; HDL-C, high-density lipoprotein cholesterol; LDL-C, low-density lipoprotein cholesterol; LVEF, left ventricular ejection fraction.^

^Normally distributed continuous variables are presented as mean ± standard deviation (SD), non-normally distributed continuous variables as median [interquartile range (IQR)], and categorical variables as count (percentage). For between-group comparisons in the unweighted cohort: one-way analysis of variance (ANOVA) was used for normally distributed continuous variables, the Kruskal-Wallis H test for non-normally distributed continuous variables, and Pearson’s Chi-square test or Fisher’s exact test for categorical variables, as appropriate^.

### Association between joint trajectories and NACE

**Figure 3:**
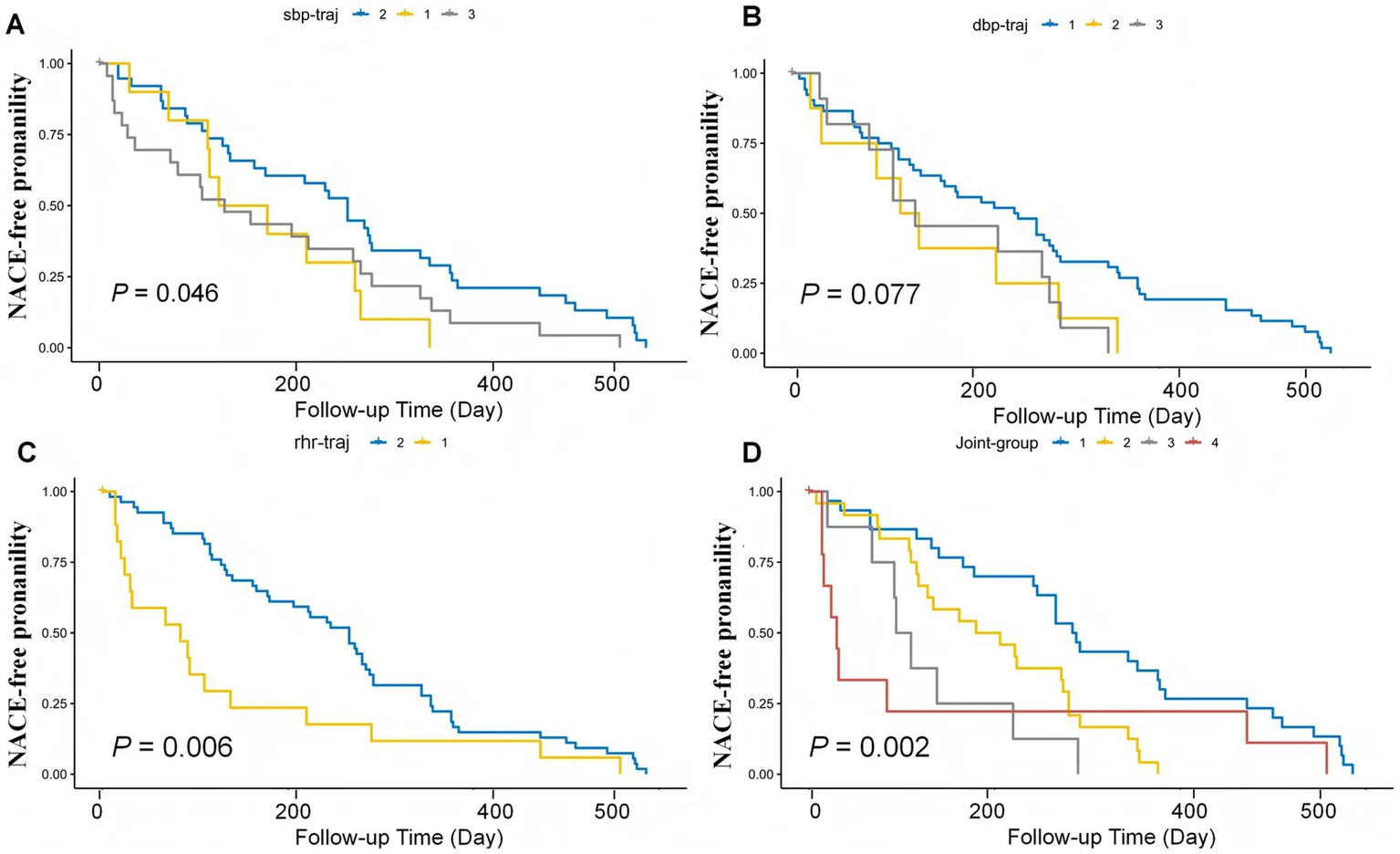
Kaplan–Meier curves for NACE-free survival across isolated and joint trajectory groups after inverse probability of treatment weighting (IPTW). **(A)** SBP trajectories; **(B)** DBP trajectories; **(C)** RHR trajectories; **(D)** Joint BP-RHR trajectories. Kaplan–Meier curves demonstrating the cumulative event-free survival rates for net adverse clinical events (NACE) stratified by systolic blood pressure (SBP), diastolic blood pressure (DBP), resting heart rate (RHR), and their joint trajectory patterns. All survival curves and corresponding log-rank *p* values were estimated based on the weighted pseudo-population after inverse probability of treatment weighting (IPTW) to rigorously balance baseline confounders.

Table 2 presents the crude and IPTW-adjusted associations between SBP, DBP, RHR, and joint trajectories with the risk of NACE. In the unweighted models, abnormal SBP, RHR, and joint trajectories were significantly associated with elevated NACE risks, whereas DBP trajectories only demonstrated a non-significant trend (both *p* > 0.05). After rigorous baseline balancing via propensity score weighting (IPTW Model), SBP and RHR trajectories remained independent predictors of NACE. Specifically, the IPTW-adjusted HRs (95% CIs) for SBP trajectories were 2.36 (1.24–4.50) for Group 1 and 1.75 (1.02–3.02) for Group 3, compared to the stable Group 2. For RHR, the fluctuating Group 1 had an IPTW HR of 1.52 (1.05–2.21). Conversely, the prognostic impact of DBP trajectories was completely attenuated toward the null after weighting (HRs approaching 1.0, both *p* > 0.40). Notably, the integration of these parameters revealed profound risk stratification: compared with the Double Stable group (Reference), the IPTW-adjusted HRs for incident NACE were 2.21 (1.23–3.97) for Group 2, 1.85 (1.09–3.13) for Group 3, and peaking at 2.69 (1.18–6.14) for the Double Fluctuating group (Group 4). Kaplan–Meier analysis further confirmed these significant prognostic differences across the isolated and joint trajectory patterns (Figure 3).

**Table 2:** Association of SBP, DBP, resting heart rate (RHR) and joint trajectories with NACE.

Variables & groups	Unweighted crude cox model				IPTW-adjusted cox model	
	*N*	Events	HR (95% CI)	*p* value	IPTW HR (95% CI)	*p* value
SBP Trajectories						
Group 2 (Ref)	437	31	1.00 (Reference)	—	1.00 (Reference)	—
Group 1	102	15	2.16 (1.11–4.22)	0.035	2.36 (1.24–4.50)	0.009
Group 3	197	25	1.75 (1.03–2.99)	0.039	1.75 (1.02–3.02)	0.044
DBP Trajectories						
Group 1 (Ref)	456	38	1.00 (Reference)	—	1.00 (Reference)	—
Group 2	119	15	1.97 (0.91–4.25)	0.084	1.10 (0.47–2.59)	0.470
Group 3	161	18	1.85 (0.93–3.65)	0.078	1.05 (0.42–2.62)	0.572
RHR Trajectories						
Group 2 (Ref)	544	46	1.00 (Reference)	—	1.00 (Reference)	—
Group 1	192	25	2.15 (1.23–3.74)	0.007	1.52 (1.05–2.21)	0.043
Joint BP-RHR Trajectories						
Group 1 (Ref)	329	20	1.00 (Reference)	—	1.00 (Reference)	—
Group 2	215	26	2.24 (1.24–4.04)	0.008	2.21 (1.23–3.97)	0.008
Group 3	108	11	2.40 (1.12–5.17)	0.025	1.85 (1.09–3.13)	0.041
Group 4	84	14	3.82 (1.65–8.87)	0.002	2.69 (1.18–6.14)	0.019

SBP, systolic blood pressure; DBP, diastolic blood pressure; RHR, resting heart rate; HR, hazard ratio; CI, confidence interval; IPTW, inverse probability of treatment weighting.

The unweighted model was evaluated using crude Cox proportional hazards regression without adjustment. The IPTW model was weighted by propensity scores to balance baseline characteristics across trajectory groups. Weights were calculated using the following baseline covariates: age, sex, body mass index, smoking, drinking, diabetes mellitus, stroke, heart failure, arrhythmia, ejection fraction, multivessel disease, and the use of β-blockers, ACEI/ARBs, and statins, as well as baseline creatinine, LDL-C, and hemoglobin levels. Robust standard errors were applied in the IPTW Cox models to estimate the 95% CIs and *p* values.

### Incremental predictive value and RCS analysis

**Figure 4:**
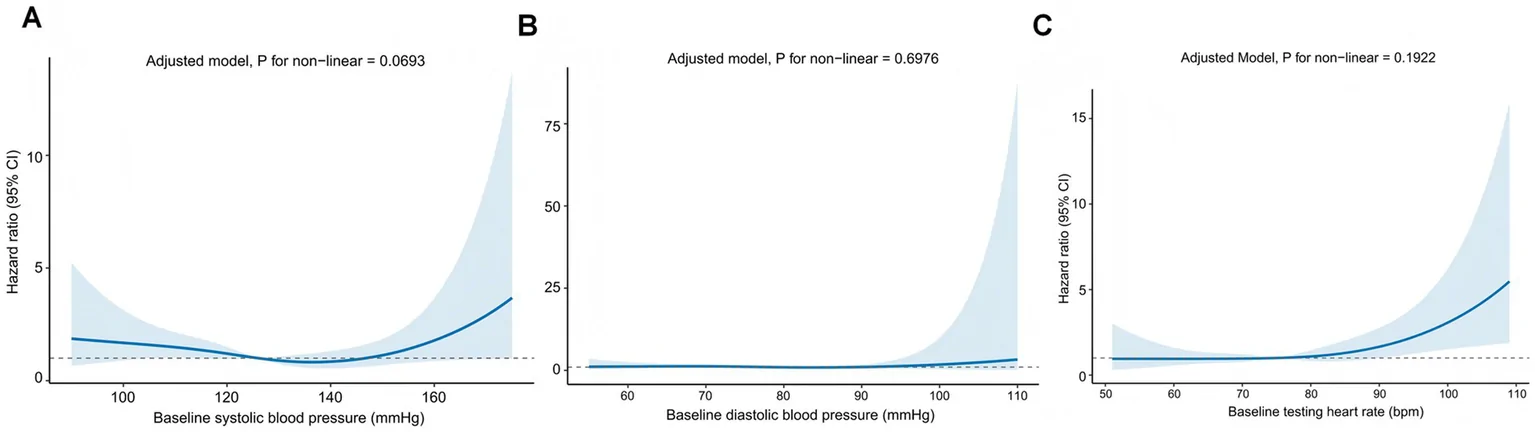
Restricted cubic spline (RCS) curves for the associations between single baseline hemodynamic parameters and the risk of the primary clinical outcome. **(A)** Baseline systolic blood pressure (SBP) showed a marginally non-linear, U-shaped trend with the outcome. **(B)** Baseline diastolic blood pressure (DBP) demonstrated a relatively flat trend with no significant non-linear association. **(C)** Baseline resting heart rate (RHR) exhibited a stable trend within the normal range and lacked statistical significance for non-linearity. Data are presented as hazard ratios (*HRs*) and 95% confidence intervals (*CIs*), represented by the solid lines and shaded areas, respectively. To facilitate clinical interpretation in alignment with standard guidelines, the reference values (where *HR* = 1.0) were predetermined at 120 mmHg for SBP, 80 mmHg for DBP, and 70 bpm for RHR. The fully adjusted Cox proportional hazards model was consistent with the multivariable analysis (Model 3), adjusting for age, sex, initial clinical presentation, diabetes mellitus, heart failure, number of LM stents, use of *β*-blockers, use of ACEI/ARB, white blood cell count, and triglycerides.

The RCS analysis demonstrated no significant non-linear associations between baseline SBP (P_non-linear_ = 0.0693), DBP (P_non-linear_ = 0.6976), or RHR (P_non-linear_ = 0.1922) and the risk of NACE (Figure 4). However, incorporating joint trajectory groups into the base model significantly enhanced discriminative performance, with the C-index increasing from 0.6779 to 0.7426 (*p* < 0.001; Table 3). This was accompanied by superior risk reclassification, as evidenced by significant integrated discrimination improvement (IDI: 0.139, *p* = 0.033), continuous net reclassification improvement (NRI: 0.463, *p* = 0.049), and median improvement (0.090, *p* = 0.048). These results highlight the incremental prognostic information provided by joint hemodynamic trajectories over conventional one-time baseline measurements.

**Table 3:** Incremental predictive value of joint BP-RHR trajectory groups for NACE.

Metrics	Base model	Full model	Difference (95% CI)	*p* value
C-index	0.6779	0.7426	0.0647 (0.0121–0.0903)	< 0.001
IDI	—	—	0.139 (0.020–0.199)	0.033
Continuous NRI	—	—	0.463 (0.002–0.593)	0.049
Median Improvement	—	—	0.090 (0.009–0.200)	0.048

C-index, concordance index; IDI, integrated discrimination improvement; NRI, net reclassification improvement; CI, confidence interval.

Base Model includes the strictly selected clinical risk factors from the multivariable Cox model: age, sex, initial clinical presentation (acute coronary syndrome vs. chronic coronary syndrome), diabetes mellitus, heart failure, number of stents in the left main (LM) coronary artery, use of β-blockers, use of ACEI/ARB, white blood cell (WBC) count, triglycerides, and additionally incorporates single baseline measurements of SBP, DBP, and RHR (as continuous variables).

Full Model includes all variables in the Base Model plus the newly identified Joint BP-RHR Trajectory Groups.

The C-index represents the model’s overall discriminative ability. The difference in C-index (ΔC-index) evaluates the incremental discrimination, with its 95% CI and *p* value calculated using the normal approximation method for correlated survival data.

IDI and Continuous NRI (cNRI) assess the improvement in risk reclassification when adding the joint trajectory groups to the baseline model, without relying on arbitrary pre-specified risk thresholds.

Median Improvement is defined as the median increase in the model-predicted probability of the endpoint event for patients who actually experienced the event, minus the median change in predicted probability for those who did not experience the event.

To prevent optimistic estimation and correct for potential overfitting in a single-center cohort, the 95% CIs and *p* values for IDI, cNRI, and Median Improvement were rigorously generated using bootstrap resampling with 1,000 iterations for internal validation.

### Subgroup and sensitivity analyses

Subgroup analyses based on the IPTW models demonstrated that the association between the high-risk joint trajectories and NACE was generally consistent across most clinical strata, including sex, diabetes, smoking status, clinical presentation, and *β*-blocker use. Nominally significant interactions were observed for heart failure status (*P* for interaction = 0.038). Furthermore, the robustness of our primary IPTW findings was thoroughly validated through multiple sensitivity analyses. The results remained consistent when evaluated using a traditional unweighted multivariable Cox proportional hazards model (Supplementary Table 9). Additionally, the high-risk trajectory groups maintained a robust association with NACE risk when additionally adjusting for baseline continuous SBP, DBP, and RHR to minimize residual confounding (Sensitivity Analysis 1), or when excluding patients with extreme baseline SBP outliers (Sensitivity Analysis 2) (Supplementary Tables 10, 11). All sensitivity analyses yielded highly consistent effect estimates for the association between the Double Fluctuating trajectory and NACE risk, with IPTW-adjusted HRs ranging from 2.58 to 2.85 (all *p* < 0.05), confirming the stability of the primary results.

## Discussion

In this prospective longitudinal study of patients undergoing PCI, we identified distinct trajectories of SBP, DBP, and RHR during the 6-month landmark period. To the best of our knowledge, this is among the early studies evaluating joint BP-RHR trajectories after PCI; in this cohort, trajectory grouping provided additional prognostic information for NACE compared with individual parameters or single baseline measurements. Specifically, compared with the Double Stable reference group, the Double Fluctuating group was associated with an increased risk of NACE in the fully IPTW-adjusted analysis (HR: 2.69, 95% CI: 1.18–6.14). The incremental predictive value was further confirmed by significant improvements in the C-index, NRI, and IDI. These findings suggest that long-term hemodynamic stability is a critical, yet often overlooked, determinant of post-PCI prognosis and may inform more refined risk stratification strategies.

Accumulating evidence indicates that blood pressure and RHR are closely associated with the postoperative prognosis of patients undergoing PCI (14, 31, 32). For example, a large population-based cohort study demonstrated a significant J-shaped relationship between baseline blood pressure and all-cause mortality in patients who underwent PCI, emphasizing the prognostic importance of optimal blood pressure control (33). Similarly, a 10-year follow-up registry (the CORFCHD-PCI study) confirmed that an elevated RHR is a strong independent predictor of major adverse cardiovascular events and mortality following PCI (34). Furthermore, studies utilizing the shock index (the ratio of RHR to systolic blood pressure) have found that integrating these two parameters serves as one of the strongest predictors of in-hospital and mortality after primary PCI (35). However, existing research primarily relies on single-time-point assessments, which fail to capture the inherent temporal volatility of these indices. Such static analytical models cannot fully represent the longitudinal trajectories of patients, thereby limiting their predictive value and clinical applications. To address this critical issue, there is an urgent need for innovative dynamic modeling frameworks that can identify early deterioration trends and facilitate real-time treatment adjustments.

The lack of independent prognostic significance for DBP trajectories is likely multifactorial, and several interrelated reasons may account for this observation. First, it may suggest that DBP has limited independent prognostic value in the contemporary post-PCI population after rigorous confounding control. This is consistent with the well-recognized collinearity between DBP and SBP. Once the dominant effect of systolic blood pressure is accounted for, the residual contribution of DBP becomes minimal. Large-scale evidence has consistently demonstrated that systolic blood-pressure elevation exerts a greater effect on cardiovascular outcomes than diastolic elevation (36), and that systolic pressure confers greater risk for heart failure than diastolic pressure (37). Second, the relationship between DBP and cardiovascular outcomes is complex and nonlinear, particularly in patients with established coronary artery disease. A well-documented J-shaped association has been observed, whereby particularly low DBP levels are associated with increased cardiovascular risk, especially in patients with established coronary artery disease (38). In the post-PCI population, this association appears to be primarily driven by extreme low DBP values (<50 mmHg), while DBP levels within the conventional range may not confer independent prognostic information after rigorous adjustment (16). Moreover, the prognostic significance of DBP appears to vary substantially across different clinical subgroups and age strata (39), which may contribute to the null findings in our overall cohort analysis. These complexities suggest that the prognostic value of DBP is highly context-dependent and may not be adequately captured by simple trajectory classification alone. Future studies in larger cohorts are warranted to further explore the prognostic value of DBP in specific patient subsets.

Consequently, our joint BP-RHR trajectory model attempts to capture the longitudinal, synergistic fluctuations of these vital signs during the vulnerable remodeling phase following PCI. Compared with traditional single time-point assessments, which merely provide a static “snapshot” and inevitably overlook inherent temporal volatility, our dynamic framework effectively quantifies the cumulative hemodynamic burden over time. Furthermore, evaluating blood pressure or RHR in isolation ignores their intricate physiological coupling. For instance, research by White et al. demonstrated that the simultaneous elevation of both parameters synergistically amplifies myocardial oxygen demand and cardiovascular mortality, representing a risk magnitude far exceeding that of either parameter alone (40). Similarly, extensive studies utilizing the shock index (the ratio of RHR to systolic blood pressure) have consistently confirmed that the mathematical integration of these vital signs yields a significantly stronger predictor of major adverse cardiovascular events following PCI than isolated blood pressure or RHR values (41). Consequently, assessing these dynamic indices separately inherently underestimates the true cumulative hemodynamic burden and risks misclassifying highly vulnerable patients. By integrating both parameters, this joint trajectory model overcomes the limitations of single-node indices, thereby providing a far more precise tool for early risk stratification and the timely optimization of secondary prevention strategies.

In addition, a major clinical insight derived from this joint approach is the identification of the “Double Fluctuating” phenotype as the primary driver of excess cardiovascular risk. Clinically, this unstable pattern may represent a higher-risk patient profile. The simultaneous and persistent fluctuation of both parameters may reflect suboptimal hemodynamic control or poor medication adherence during the critical post-discharge period, as well as potential refractory response to guideline-directed medical therapy. Several potential pathophysiological mechanisms may underpin the observed association between this joint trajectory phenotype and increased NACE risk. Rather than acting through a single pathway, this phenotype could impose a detrimental “double hit” on the cardiovascular system, though these mechanistic inferences remain hypothesis-generating given the observational design of our study (42–44). First, from a biomechanical perspective, fluctuating systolic blood pressure may increase afterload and pathological shear stress on the stented vascular segment, potentially accelerating in-stent neoatherosclerosis (45, 46). Concurrently, elevated resting heart rate may disproportionately shorten diastolic coronary perfusion, creating a supply–demand mismatch that could compromise myocardial energetics (47). Finally, chronic hemodynamic instability may be associated with sustained autonomic nervous system dysfunction and sympathetic overdrive, which could promote a pro-inflammatory and pro-thrombotic state that predisposes to ischemic events or heart failure (48). These mechanistic pathways could serve as hypotheses for future mechanistic studies.

Interestingly, our exploratory subgroup analyses revealed nominally significant interactions, with the prognostic impact of the “Double Fluctuating” phenotype appearing more pronounced in those without pre-existing HF. While this might suggest that severe hemodynamic fluctuations serve as a clearer early warning signal in patients without the competing risks of advanced age or established structural remodeling (where intensive polypharmacy may blunt physiological signals), these mechanistic inferences should not be overstated (49, 50). Importantly, given the exploratory nature of these tests, the limited event counts within specific strata, and the lack of formal adjustment for multiple comparisons, these interactions must be interpreted with extreme caution and considered strictly hypothesis-generating (51, 52). Nevertheless, the high-risk joint trajectory consistently maintained its overarching predictive validity across the vast majority of patient strata, reinforcing its broad applicability in general post-PCI risk stratification (53).

## Strengths and limitations

As easily accessible, non-invasive, and serially measurable clinical parameters, joint blood pressure and RHR trajectory monitoring offers two primary clinical advantages for post-PCI management: (1) Cost-effective early risk stratification: By utilizing routine outpatient vital signs without incurring additional medical expenses, clinicians can readily identify highly vulnerable patients—particularly those exhibiting the high-risk “Double Fluctuating” phenotype. (2) Optimization of personalized secondary prevention: Recognizing these unstable longitudinal trajectories enables physicians to implement timely, targeted interventions. For these high-risk patients, clinicians can initiate the aggressive titration of cardioprotective medications, alongside stringent medication adherence monitoring and lifestyle counseling, to ultimately reduce the incidence of NACE and improve clinical outcomes.

Despite these significant clinical implications, this study has several limitations that should be explicitly acknowledged. First, given the observational nature of our research, although we rigorously adjusted for key clinical, laboratory, and procedural confounders in the multivariable regression models, the potential for residual or unmeasured confounding cannot be entirely excluded. Second, outpatient blood pressure and RHR levels may be transiently influenced by various dynamic factors, such as the white-coat effect, concurrent psychological stress, the precise timing of medication administration relative to the clinic visit, and natural diurnal variations, which might introduce episodic measurement bias. Third, we note that the loss to follow-up rate was relatively high, although baseline characteristics were comparable between included and excluded participants. Moreover, as a single-center study lacking an external validation cohort, the broad generalizability and reproducibility of our joint trajectory models across different ethnic populations and disparate healthcare systems remain inherently limited. Finally, while the joint trajectory model demonstrated robust prognostic value, its clinical utility in actively guiding targeted therapeutic interventions has not been prospectively validated. Several subgroup analyses involved small sample sizes and limited numbers of events, which may reduce statistical precision. These findings should therefore be interpreted as exploratory and hypothesis-generating. Therefore, while our findings are exploratory and hypothesis-generating, the joint monitoring of blood pressure and RHR trajectories may represent a promising, cost-effective tool for risk stratification in post-PCI patients. These results suggest that actively optimizing these dynamic hemodynamic profiles could potentially help reduce the incidence of NACEs and improve clinical outcomes. However, given the observational design and the lack of external validation, these findings warrant confirmation in future prospective multicenter studies and randomized controlled trials before any definitive recommendation can be made to integrate trajectory-guided management into routine secondary prevention strategies for post-PCI care.

## Conclusion

In conclusion, this prospective longitudinal study demonstrates that joint 6-month blood pressure and resting heart rate trajectories are independently associated with the risk of net adverse clinical events in patients following PCI. The incorporation of these dynamic joint trajectories significantly improves the discriminative performance of conventional risk stratification models compared with static single-point measurements. Routine monitoring of joint hemodynamic trajectories may serve as a cost-effective reference tool for post-PCI risk stratification and may assist in the individualized refinement of secondary prevention strategies.

## Data Availability

The raw data supporting the conclusions of this article will be made available by the authors, without undue reservation.

## Glossary

ACEI/ARB: angiotensin-converting enzyme inhibitors/angiotensin II receptor blockers;
ACS: acute coronary syndrome
AMI: acute myocardial infarction
BMI: body mass index
BP: blood pressure
CCS: chronic coronary syndrome
CEC: clinical event committee
CI: confidence interval
C-index: concordance index
cNRI: continuous net reclassification improvement
Cr: creatinine
DBP: diastolic blood pressure
DM: diabetes mellitus
EF: ejection fraction
EMR: electronic medical records
EPV: events per variable
FBG: fasting blood glucose
Hb: hemoglobin
HbA1c: glycosylated hemoglobin
HDL-C: high-density lipoprotein cholesterol
HF: heart failure
HR: hazard ratio
IDI: integrated discrimination improvement
IQR: interquartile range
IPTW: inverse probability of treatment weighting
LAD: left anterior descending artery
LCX: left circumflex artery
LCTM: latent class trajectory modeling
LDL-C: low-density lipoprotein cholesterol
LM: left main coronary artery
LVEF: left ventricular ejection fraction
NACE: net adverse clinical events
NYHA: New York Heart Association
PCI: percutaneous coronary intervention
PLT: platelets
RCA: right coronary artery
RCS: restricted cubic spline
RHR: resting heart rate
SBP: systolic blood pressure
SD: standard deviation
SMD: standardized mean difference
TG: triglycerides
WBC: white blood cell
