# Supplementary material for: Joint blood pressure and resting heart rate trajectories and net adverse clinical events risk following PCI: an inverse probability of treatment weighting analysis
Source: Front Med (Lausanne). 2026 Sep 7;13:1914874. doi: 10.3389/fmed.2026.1914874 (PMC13590288; doi:10.3389/fmed.2026.1914874)
Supplement: Supplementary file 1 [file Supplementary_file_1.docx]

**Supplementary Materials**

**Joint longitudinal trajectories of blood pressure and resting heart rate and the risk of net adverse clinical events following PCI: an inverse probability of treatment weighting analysis**

**Supplementary Tables**

**Supplementary Table 1. Model fit indices for latent class trajectory modeling of SBP, DBP, and RHR.** Values represent model fit statistics BIC, AIC, Entropy and minimum class size (%). Latent class trajectory modeling was performed to identify optimal trajectory classes for each hemodynamic parameter, with model selection based on lower BIC/AIC, Entropy >0.70, average posterior probability ≥0.70, and minimum class size >5%. AIC, Akaike Information Criterion; BIC, Bayesian Information Criterion; DBP, diastolic blood pressure; NA, not applicable; RHR, resting heart rate; SBP, systolic blood pressure.

**Supplementary Table 2. Average posterior probabilities of the latent class trajectory models for SBP, DBP, and RHR. .**Data are presented as average posterior probability (APP) for each latent class. APP ≥0.70 indicates acceptable classification reliability. APP, average posterior probability; BIC, Bayesian Information Criterion; DBP, diastolic blood pressure; RHR, resting heart rate; SBP, systolic blood pressure.

**Supplementary Table 3. Baseline characteristics of patients stratified by SBP trajectory classes.** Values represent mean ± standard deviation for continuous variables or count (percentage) for categorical variables. Comparisons were performed using one-way ANOVA/Kruskal-Wallis test for continuous variables and Chi-square test/Fisher’s exact test for categorical variables. ACEI/ARB, angiotensin-converting enzyme inhibitors/angiotensin II receptor blockers; BMI, body mass index; DM, diabetes mellitus; DBP, diastolic blood pressure; EF, ejection fraction; FBG, fasting blood glucose; Hb, hemoglobin; HbA1c, glycated hemoglobin; HDL-C, high-density lipoprotein cholesterol; HF, heart failure; IQR, interquartile range; LCX, left circumflex; LAD, left anterior descending; LDL-C, low-density lipoprotein cholesterol; LM, left main; NYHA, New York Heart Association; PLT, platelet; RCA, right coronary artery; SD, standard deviation; SBP, systolic blood pressure; TG, triglycerides; WBC, white blood cell.

**Supplementary Table 4. Baseline characteristics of patients stratified by DBP trajectory classes.** Values represent mean ± standard deviation for continuous variables or count (percentage) for categorical variables. Comparisons were conducted using one-way ANOVA/Kruskal-Wallis test for continuous variables and Chi-square test/Fisher’s exact test for categorical variables. ACEI/ARB, angiotensin-converting enzyme inhibitors/angiotensin II receptor blockers; BMI, body mass index; DM, diabetes mellitus; DBP, diastolic blood pressure; EF, ejection fraction; FBG, fasting blood glucose; Hb, hemoglobin; HbA1c, glycated hemoglobin; HDL-C, high-density lipoprotein cholesterol; HF, heart failure; IQR, interquartile range; LCX, left circumflex; LAD, left anterior descending; LDL-C, low-density lipoprotein cholesterol; LM, left main; NYHA, New York Heart Association; PLT, platelet; RCA, right coronary artery; SD, standard deviation; TG, triglycerides; WBC, white blood cell.

**Supplementary Table 5. Baseline characteristics of patients stratified by RHR trajectory classes.** Values represent mean ± standard deviation for continuous variables or count (percentage) for categorical variables. Group comparisons were performed using Student’s t-test/Mann-Whitney U test for continuous variables and Chi-square test/Fisher’s exact test for categorical variables. ACEI/ARB, angiotensin-converting enzyme inhibitors/angiotensin II receptor blockers; BMI, body mass index; DM, diabetes mellitus; EF, ejection fraction; FBG, fasting blood glucose; Hb, hemoglobin; HbA1c, glycated hemoglobin; HDL-C, high-density lipoprotein cholesterol; HF, heart failure; IQR, interquartile range; LCX, left circumflex; LAD, left anterior descending; LDL-C, low-density lipoprotein cholesterol; LM, left main; NYHA, New York Heart Association; PLT, platelet; RCA, right coronary artery; RHR, resting heart rate; SD, standard deviation; TG, triglycerides; WBC, white blood cell.

**Supplementary Table 6. Baseline characteristics of Included vs. Excluded Patients.** Values represent mean ± standard deviation for continuous variables or count (percentage) for categorical variables. Comparisons were made using Student’s t-test/Mann-Whitney U test for continuous variables and Chi-square test/Fisher’s exact test for categorical variables. ACEI/ARB, angiotensin-converting enzyme inhibitors/angiotensin II receptor blockers; ACS, acute coronary syndrome; BMI, body mass index; CCS, chronic coronary syndrome; DM, diabetes mellitus; EF, ejection fraction; FBG, fasting blood glucose; Hb, hemoglobin; HbA1c, glycated hemoglobin; HDL-C, high-density lipoprotein cholesterol; HF, heart failure; IQR, interquartile range; LCX, left circumflex; LAD, left anterior descending; LDL-C, low-density lipoprotein cholesterol; LM, left main; NYHA, New York Heart Association; PLT, platelet; RCA, right coronary artery; SD, standard deviation; TG, triglycerides; WBC, white blood cell.

**Supplementary Table 7**. Baseline demographic, clinical, and laboratory characteristics of study participants stratified by combined blood pressure and resting heart rate control status in the unweighted and IPTW-weighted cohorts

**Supplementary Table 8. Actual SBP, DBP, and RHR values at baseline and follow-up visits stratified by joint trajectory classes.** Values represent mean ± standard deviation (mmHg for BP, bpm for RHR). Longitudinal hemodynamic measurements were collected at baseline (Month 0), 1-month, 3-month, and 6-month follow-up visits. bpm, beats per minute; DBP, diastolic blood pressure; RHR, resting heart rate; SBP, systolic blood pressure; SD, standard deviation.

**Supplementary Table 9. Association of SBP, DBP, RHR, and Joint Trajectories with Risk of Cardiovascular Events.** Values represent hazard ratio (HR) and 95% confidence interval (CI). Cox proportional hazards regression models were progressively adjusted: Model 1 (age, sex); Model 2 (Model 1 + clinical presentation, DM, HF); Model 3 (Model 2 + LM stents, β-blockers, ACEI/ARB, WBC count, TG). P for trend was calculated by entering trajectory groups as a continuous variable. CI, confidence interval; DBP, diastolic blood pressure; HR, hazard ratio; RHR, resting heart rate; SBP, systolic blood pressure.

**Supplementary Table 10. Subgroup analyses for the association between the high-risk joint trajectory and the risk of clinical outcomes.** Values represent inverse probability of treatment weighting (IPTW)-adjusted HR and 95% CI. Analyses were stratified by clinical characteristics, with P for interaction derived from likelihood ratio tests. IPTW-adjusted HR compares the Double Fluctuating group vs. Double Stable group within each stratum. ACS, acute coronary syndrome; CI, confidence interval; CCS, chronic coronary syndrome; DM, diabetes mellitus; HF, heart failure; HR, hazard ratio; IPTW, inverse probability of treatment weighting.

**Supplementary Table 11. Sensitivity analyses for the associations between blood pressure, RHR Trajectories, and clinical outcomes.** Values represent IPTW-adjusted HR and 95% CI. Sensitivity Analysis 1: Adjusted for baseline SBP, DBP, and RHR as continuous variables. Sensitivity Analysis 2: Excluded patients with extreme baseline SBP outliers. CI, confidence interval; DBP, diastolic blood pressure; HR, hazard ratio; IPTW, inverse probability of treatment weighting; RHR, resting heart rate; SBP, systolic blood pressure.

**Supplementary Figures**

**Supplementary Figure 1. Individual observed versus predicted mean longitudinal trajectories for the three latent classes of diastolic blood pressure.** (A) Class 1: High-decreasing-increasing; (B) Class 2: Stable; (C) Class 3: Low-increasing. The bold solid lines represent the model-predicted mean trajectories for that specific class. The lighter, transparent lines represent the actual observed longitudinal measurements of individual patients assigned to the respective class based on their highest posterior probability. The figure illustrates the goodness-of-fit of the DBP trajectory model over the 6-month follow-up period.

**Supplementary Figure 2. Individual observed versus predicted mean longitudinal trajectories for the three latent classes of systolic blood pressure.**  (A) Class 1: High-decreasing-increasing; (B) Class 2: Stable; (C) Class 3: Low-increasing. The bold solid lines represent the model-predicted mean trajectories for that specific class. The lighter, transparent lines represent the actual observed longitudinal measurements of individual patients assigned to the respective class based on their highest posterior probability. The figure demonstrates the goodness-of-fit of the SBP trajectory model over the 6-month follow-up period.

**Supplementary Figure 3. Individual observed versus predicted mean longitudinal trajectories for the two latent classes of resting heart rate.** (A) Class 1: Fluctuating; (B) Class 2: Stable. Bold solid lines show model-predicted mean trajectories, and lighter transparent lines represent individual patients’ observed longitudinal measurements assigned to the class based on highest posterior probability. The figure shows the goodness-of-fit of the RHR trajectory model over the 6-month follow-up period.

**Latent class trajectory modeling (LCTM)**

Latent Class Trajectory Modeling (LCTM)To identify distinct longitudinal patterns of SBP, DBP, and RHR over the follow-up period, we employed Latent Class Trajectory Modeling (LCTM) using the hlme function in R. To account for missing repeated measurements over time, models were estimated using full information maximum likelihood (FIML) under the missing-at-random assumption, which utilizes all available data without excluding patients with incomplete longitudinal records.For each parameter (SBP, DBP, and RHR), we systematically tested models ranging from 1 to 6 latent classes. The trajectory shapes were flexibly specified by evaluating different polynomial functional forms (including linear, quadratic, and cubic terms) to best capture the temporal dynamics. To ensure model parsimony, prevent overfitting, and achieve model convergence, within-class random effects were constrained to zero (i.e., a Latent Class Growth Analysis framework).The optimal number of classes for each parameter was determined based on a comprehensive set of statistical and clinical criteria: (1) lower values of the Bayesian Information Criterion (BIC) and Akaike Information Criterion (AIC); (2) an Entropy value closer to 1 (typically >0.70), indicating excellent overall class separation; (3) an average posterior probability (APP) 0.70 for all classes, confirming high internal reliability of individual assignments; and (4) a minimum class size of >5% to ensure adequate statistical power for subsequent survival analyses.Following the independent identification of the optimal models for SBP (3 classes), DBP (3 classes), and RHR (2 classes), individuals were assigned to their most likely trajectory class based on the maximum posterior probability. The specific model fit indices (BIC, AIC, Entropy, minimum class size) and the APPs for all 1-to-6 class models are exhaustively detailed in Supplementary Table 1 and Supplementary Table 2, respectively. Finally, a joint trajectory grouping strategy was implemented by cross-tabulating the individual assignments from the isolated SBP, DBP, and RHR models. Based on clinical overlapping and sample size feasibility, these intersecting categories were ultimately consolidated into 4 distinct joint BP-RHR trajectory groups (Supplementary Tables 1-2).

**Incremental predictive value analysis**

Incremental predictive value analysis To evaluate whether the joint BP-RHR trajectory groups provided incremental prognostic value beyond established clinical risk factors and conventional single baseline hemodynamic measurements, we calculated the change in Harrell’s C-index (ΔC-index), the continuous Net Reclassification Improvement (cNRI), and the Integrated Discrimination Improvement (IDI). Specifically, the baseline clinical model (Base Model) was rigorously defined using the covariates from our fully adjusted parsimonious Cox model (Model 3), which included age, sex, initial clinical presentation (acute coronary syndrome vs. chronic coronary syndrome), diabetes mellitus, heart failure, number of stents in the left main (LM) coronary artery, use of β-blockers, use of ACEI/ARB, white blood cell (WBC) count, and triglycerides. **To more rigorously test the incremental value of longitudinal trajectory patterns beyond static snapshot assessments, the Base Model was further adjusted for baseline single-measurement SBP, DBP, and RHR as continuous variables.** The updated full model (Full Model) was constructed by adding the joint BP-RHR trajectory group variable to this predefined Base Model. Furthermore, to mitigate the risk of optimistic performance estimates and potential overfitting inherent in a single-center cohort, we performed rigorous internal validation. The 95% confidence intervals (CIs) for the ΔC-index, cNRI, and IDI were obtained using bootstrap resampling with 1,000 iterations. We utilized continuous NRI rather than categorical NRI, as it does not rely on arbitrary pre-specified risk thresholds, which is more appropriate for our specific study cohort. The statistical significance of the difference in C-index between the two models was tested using DeLong’s method

**Traditional multivariable cox proportional hazards regression**

To rigorously validate the robustness of our primary findings derived from the inverse probability of treatment weighting pseudo population, we conducted a pre specified sensitivity analysis using traditional progressively adjusted multivariable Cox proportional hazards regression models in the original unweighted cohort. This dual validation approach ensures that our results are not overly reliant on a single statistical framework and provides familiar interpretability for clinical practice. Given the total number of 71 endpoint events, we meticulously selected covariates based on established clinical relevance and significant baseline imbalances, strictly adhering to the reliable threshold of approximately 5 to 10 events per variable to avoid statistical overfitting. Three progressive models were constructed to evaluate the independent association between the joint blood pressure and RHR Trajectories and the risk of net adverse clinical events. Model 1 was adjusted for baseline demographic characteristics including age and sex. Model 2 was further adjusted for initial clinical presentation, diabetes mellitus, and baseline heart failure. The fully adjusted model, referred to as Model 3, additionally incorporated critical procedural features, discharge medications, and essential laboratory findings that demonstrated significant variations across groups, specifically the number of stents in the left main coronary artery, the use of beta blockers, angiotensin converting enzyme inhibitors or angiotensin receptor blockers, white blood cell count, and triglycerides. Multicollinearity among the adjusted covariates was assessed using the variance inflation factor, and all values were strictly below 5.0, indicating the absence of significant collinearity. The consistency of hazard ratios across both the main weighted model and these traditional multivariable models further reinforces the independent prognostic value of the double fluctuating phenotype.

**Subgroup and Sensitivity Analyses**

To evaluate the consistency of the prognostic impact of the high-risk joint trajectory across various clinical profiles, we conducted exploratory subgroup analyses. Patients were stratified by age (< 65 vs. ≥ 65 years), sex, diabetes mellitus, smoking status, baseline heart failure, clinical presentation (ACS vs. CCS), and the use of $\beta$-blockers. Within each stratum, the hazard ratios were estimated, and potential effect modifications were evaluated by introducing interaction terms into the Cox models.

Crucially, given the overall limited number of primary endpoint events (n=71), we prespecified that these subgroup analyses were strictly exploratory and hypothesis-generating. To ensure complete transparency regarding statistical power, the exact number of events and total patients within each stratum were explicitly reported. *P*-values for interaction were considered nominal, as no formal adjustments were made for multiple comparisons. Consequently, any nominally significant interactions (e.g., age and heart failure) were interpreted with extreme caution, avoiding overly definitive mechanistic inferences.

Furthermore, two rigorous sensitivity analyses were performed to verify the robustness of our primary Cox models: (1) additionally adjusting for baseline SBP, DBP, and RHR as continuous variables to rule out residual confounding from initial vital signs; and (2) excluding patients with extreme baseline SBP outliers to ensure that the defined trajectory patterns were not driven by extreme initial values.

**Supplementary Table 1**. Model fit indices for latent class trajectory modeling of SBP, DBP, and RHR^1^

| **Trajectory Variable** | **Number of classes**  **(K)** | **BIC** | **AIC** | **Entropy** | **Minimum class size**  **(%)** |
| --- | --- | --- | --- | --- | --- |
| **Systolic Blood Pressure** | 1 | 23169.83 | 23137.62 | NA | NA |
|  | 2 | 22891.03 | 22840.42 | 0.65 | 33.56 |
|  | **3** | **22833.48** | **22764.46** | **0.79** | **13.86** |
|  | 4 | 22815.47 | 22728.05 | 0.81 | 2.85 |
|  | 5 | 22834.75 | 22728.92 | 0.80 | 0.82 |
|  | 6 | 22853.76 | 22729.53 | 0.76 | 0.27 |
| **Diastolic Blood Pressure** | 1 | 20598.99 | 20566.78 | NA | NA |
|  | 2 | 20376.28 | 20325.66 | 0.63 | 30.71 |
|  | **3** | **20350.51** | **20265.49** | **0.78** | **16.17** |
|  | 4 | 20355.86 | 20268.44 | 0.77 | 0.14 |
|  | 5 | 20377.99 | 20272.16 | 0.77 | 0.14 |
|  | 6 | 20399.30 | 20275.07 | 0.75 | 0.14 |
| **Resting Heart Rate** | 1 | 21183.33 | 21151.12 | NA | NA |
|  | **2** | **20814.29** | **20753.68** | **0.79** | **26.09** |
|  | 3 | 20865.23 | 20756.21 | 0.80 | 2.17 |
|  | 4 | 20885.32 | 20767.89 | 0.69 | 1.77 |
|  | 5 | 20885.12 | 20769.29 | 0.71 | 0.27 |
|  | 6 | 20896.07 | 20771.84 | 0.57 | 1.77 |
| Abbreviations: SBP, systolic blood pressure; DBP, diastolic blood pressure; RHR, resting heart rate; BIC, Bayesian Information Criterion; AIC, Akaike Information Criterion; NA, not applicable. | | | | | |
| ^1^ Bold text indicates the optimal model selected for each parameter. The selection criteria were based on a lower Bayesian Information Criterion (BIC) and Akaike Information Criterion (AIC), an Entropy value closer to 1 (typically > 0.70 indicating good class separation), and a clinical consideration that the minimum class size should be > 5% to ensure adequate statistical power for subsequent survival analyses. | | | | | |

**Supplementary Table 2.** Average posterior probabilities of the latent class trajectory models for SBP, DBP, and RHR^1.^

| **Trajectory Variable** | **Number of classes (K)** | **BIC** | **APP 1** | **APP 2** | **APP 3** | **APP 4** | **APP 5** | **APP 6** |
| --- | --- | --- | --- | --- | --- | --- | --- | --- |
| **Systolic Blood Pressure** | 1 | 23169.83 | 1.00 | - | - | - | - | - |
|  | 2 | 22891.03 | 0.91 | 0.86 | - | - | - | - |
|  | **3** | **22833.48** | **0.84** | **0.82** | **0.80** | - | - | - |
|  | 4 | 22815.47 | 0.83 | 0.78 | 0.82 | 0.85 | - | - |
|  | 5 | 22834.75 | 0.86 | 0.76 | 0.82 | 0.79 | 0.74 | - |
|  | 6 | 22853.76 | 0.75 | 0.77 | 0.76 | 0.95 | 0.67 | 0.84 |
| **Diastolic Blood Pressure** | 1 | 20598.99 | 1.00 | - | - | - | - | - |
|  | 2 | 20376.28 | 0.86 | 0.90 | - | - | - | - |
|  | **3** | **20350.51** | **0.80** | **0.82** | **0.78** | - | - | - |
|  | 4 | 20355.86 | 0.77 | 1.00 | 0.82 | 0.80 | - | - |
|  | 5 | 20377.99 | 0.80 | 0.73 | 0.74 | 1.00 | 0.78 | - |
|  | 6 | 20399.30 | 0.81 | 1.00 | 0.82 | 0.76 | - | 1.00 |
| **Resting Heart Rate** | 1 | 21183.33 | 1.00 | - | - | - | - | - |
|  | **2** | **20814.29** | **0.87** | **0.92** | - | - | - | - |
|  | 3 | 20865.23 | 0.86 | 0.89 | 0.93 | - | - | - |
|  | 4 | 20885.32 | 0.82 | 0.76 | 0.92 | 0.85 | - | - |
|  | 5 | 20885.12 | 0.84 | 0.82 | 0.74 | 0.92 | 0.93 | - |
|  | 6 | 20896.07 | - | 0.81 | 0.47 | 0.67 | 0.70 | 0.90 |
| Abbreviations: SBP, systolic blood pressure; DBP, diastolic blood pressure; RHR, resting heart rate; BIC, Bayesian Information Criterion; APP, average posterior probability. | | | | | | | | |
| ^1^Bold text indicates the optimal model selected for each parameter based on comprehensive fit indices. The Average Posterior Probability (APP) evaluates the classification quality of the trajectory models. An APP value ≥ 0.70 generally indicates adequate internal reliability and distinct group assignment for the individuals assigned to each trajectory class. | | | | | | | | |

**Supplementary Table 3**. Baseline characteristics of patients stratified by SBP trajectory classes ^1^

| **Characteristics** | **Class 1:**  **High-decreasing-increasing (n=102)** | **Class 2: Stable(n=437)** | **Class 3: Low-increasing (n=197)** | ***P* value** |
| --- | --- | --- | --- | --- |
| **Demographics & Lifestyle** |  |  |  |  |
| Age, mean (SD) | 60.69 (10.51) | 61.26 (10.29) | 60.37 (10.21) | 0.579 |
| Sex, n (%) |  |  |  | 0.809 |
| Male | 81 (79.4) | 334 (76.4) | 151 (76.6) |  |
| Female | 21 (20.6) | 103 (23.6) | 46 (23.4) |  |
| BMI, mean (SD) | 25.47 (2.95) | 24.94 (3.14) | 24.30 (3.08) | 0.006 |
| Smoking, n (%) |  |  |  | 0.966 |
| Never | 58 (56.9) | 252 (57.7) | 108 (54.8) |  |
| Current | 34 (33.3) | 142 (32.5) | 70 (35.5) |  |
| Former | 10 (9.8) | 43 (9.8) | 19 (9.6) |  |
| Drinking, n (%) |  |  |  | 0.688 |
| Never | 83 (81.4) | 349 (79.9) | 158 (80.2) |  |
| Current | 19 (18.6) | 85 (19.5) | 36 (18.3) |  |
| Former | 0 (0.0) | 3 (0.7) | 3 (1.5) |  |
| **Medical History** |  |  |  |  |
| DM, n (%) |  |  |  | 0.497 |
| No | 73 (71.6) | 287 (65.7) | 129 (65.5) |  |
| Yes | 29 (28.4) | 150 (34.3) | 68 (34.5) |  |
| Hyperlipidemia, n (%) |  |  |  | 0.504 |
| No | 79 (77.5) | 348 (79.6) | 163 (82.7) |  |
| Yes | 23 (22.5) | 89 (20.4) | 34 (17.3) |  |
| Hyperuricemia, n (%) |  |  |  | 0.644 |
| No | 100 (98.0) | 428 (97.9) | 195 (99.0) |  |
| Yes | 2 (2.0) | 9 (2.1) | 2 (1.0) |  |
| Stroke, n (%) |  |  |  | 0.785 |
| No | 85 (83.3) | 371 (84.9) | 170 (86.3) |  |
| Yes | 17 (16.7) | 66 (15.1) | 27 (13.7) |  |
| HF, n (%) |  |  |  | 0.005 |
| No | 98 (96.1) | 406 (92.9) | 170 (86.3) |  |
| Yes | 4 (3.9) | 31 (7.1) | 27 (13.7) |  |
| NYHA functional class, n (%) |  |  |  | 0.168 |
| Normal/0 | 26 (25.5) | 104 (23.8) | 40 (20.3) |  |
| Class I | 11 (10.8) | 49 (11.2) | 24 (12.2) |  |
| Class II | 56 (54.9) | 262 (60.0) | 111 (56.3) |  |
| Class III | 9 (8.8) | 19 (4.3) | 18 (9.1) |  |
| Class IV | 0 (0.0) | 3 (0.7) | 4 (2.0) |  |
| Anemia, n (%) |  |  |  | 0.985 |
| No | 100 (98.0) | 429 (98.2) | 193 (98.0) |  |
| Yes | 2 (2.0) | 8 (1.8) | 4 (2.0) |  |
| Arrhythmia, n (%) |  |  |  | 0.655 |
| No | 91 (89.2) | 390 (89.2) | 171 (86.8) |  |
| Yes | 11 (10.8) | 47 (10.8) | 26 (13.2) |  |
| **Angiography & Interventions** |  |  |  |  |
| Left main disease, n (%) |  |  |  | 0.849 |
| No | 88 (86.3) | 380 (87.0) | 168 (85.3) |  |
| Yes | 14 (13.7) | 57 (13.0) | 29 (14.7) |  |
| LAD lesion, n (%) |  |  |  | 0.649 |
| No | 14 (13.7) | 59 (13.5) | 32 (16.2) |  |
| Yes | 88 (86.3) | 378 (86.5) | 165 (83.8) |  |
| LCX lesion, n (%) |  |  |  | 0.349 |
| No | 23 (22.5) | 130 (29.7) | 56 (28.4) |  |
| Yes | 79 (77.5) | 307 (70.3) | 141 (71.6) |  |
| RCA lesion, n (%) |  |  |  | 0.993 |
| No | 32 (31.4) | 138 (31.6) | 63 (32.0) |  |
| Yes | 70 (68.6) | 299 (68.4) | 134 (68.0) |  |
| Multivessel disease, n (%) |  |  |  | 0.195 |
| 0-vessel | 6 (5.9) | 14 (3.2) | 13 (6.6) |  |
| 1-vessel | 14 (13.7) | 80 (18.3) | 27 (13.7) |  |
| 2-vessel | 23 (22.5) | 125 (28.6) | 58 (29.4) |  |
| 3-vessel | 59 (57.8) | 218 (49.9) | 99 (50.3) |  |
| Stents in LM, mean (SD) | 0.12 (0.32) | 0.12 (0.36) | 0.12 (0.38) | >0.99 |
| Stents in LAD, mean (SD) | 0.83 (0.98) | 0.84 (0.97) | 0.89 (0.94) | 0.767 |
| Stents in LCX, mean (SD) | 0.28 (0.57) | 0.25 (0.58) | 0.21 (0.52) | 0.540 |
| Stents in RCA, mean (SD) | 0.54 (0.98) | 0.46 (0.83) | 0.45 (0.84) | 0.649 |
| Total number of stents, mean (SD) | 1.77 (1.01) | 1.67 (1.04) | 1.67 (0.96) | 0.613 |
| **Medications** |  |  |  |  |
| β-blockers, n (%) |  |  |  | 0.449 |
| No | 26 (25.5) | 135 (30.9) | 64 (32.5) |  |
| Yes | 76 (74.5) | 302 (69.1) | 133 (67.5) |  |
| ACEI/ARB, n (%) |  |  |  | 0.004 |
| No | 73 (71.6) | 358 (81.9) | 172 (87.3) |  |
| Yes | 29 (28.4) | 79 (18.1) | 25 (12.7) |  |
| Statin, n (%) |  |  |  | 0.768 |
| No | 6 (5.9) | 21 (4.8) | 12 (6.1) |  |
| Yes | 96 (94.1) | 416 (95.2) | 185 (93.9) |  |
| P2Y12 inhibitors, n (%) |  |  |  | 0.141 |
| No | 1 (1.0) | 3 (0.7) | 5 (2.5) |  |
| Yes | 101 (99.0) | 434 (99.3) | 192 (97.5) |  |
| Aspirin, n (%) |  |  |  | 0.252 |
| No | 4 (3.9) | 31 (7.1) | 18 (9.1) |  |
| Yes | 98 (96.1) | 406 (92.9) | 179 (90.9) |  |
| **Laboratory Results & Echo** |  |  |  |  |
| WBC, median [IQR] | 7.28 [6.15, 8.94] | 6.99 [5.30, 8.45] | 7.13 [5.50, 9.09] | 0.104 |
| Hb, mean (SD) | 139.96 (23.23) | 139.16 (18.19) | 135.13 (22.43) | 0.042 |
| PLT, mean (SD) | 201.49 (61.51) | 205.67 (63.04) | 211.14 (65.32) | 0.414 |
| HbA1c, mean (SD) | 6.42 (1.11) | 6.67 (1.44) | 6.60 (1.52) | 0.270 |
| FBG, mean (SD) | 6.01 (1.96) | 6.34 (2.35) | 6.37 (2.59) | 0.400 |
| K, mean (SD) | 3.94 (0.35) | 3.96 (0.37) | 4.02 (0.40) | 0.117 |
| Cr, median [IQR] | 73.0 [61.3, 97.5] | 73.0 [60.0, 102.0] | 75.0 [62.0, 122.0] | 0.470 |
| HDL_C, mean (SD) | 1.08 (0.36) | 1.06 (0.39) | 1.03 (0.36) | 0.438 |
| LDL_C, mean (SD) | 2.24 (1.01) | 2.35 (1.17) | 2.36 (1.31) | 0.646 |
| TG, median [IQR] | 3.71 [2.93, 4.73] | 3.83 [2.99, 4.61] | 3.62 [2.95, 4.58] | 0.563 |
| EF, mean (SD) | 59.39 (11.24) | 57.99 (12.40) | 57.21 (12.20) | 0.340 |
| Abbreviations: SBP, systolic blood pressure; BMI, body mass index; DM, diabetes mellitus; HF, heart failure; NYHA, New York Heart Association; LM, left main; LAD, left anterior descending; LCX, left circumflex; RCA, right coronary artery; ACEI/ARB, angiotensin-converting enzyme inhibitors/angiotensin receptor blockers; WBC, white blood cell; Hb, hemoglobin; PLT, platelet; HbA1c, glycated hemoglobin; FBG, fasting blood glucose; K, potassium; Cr, creatinine; HDL-C, high-density lipoprotein cholesterol; LDL-C, low-density lipoprotein cholesterol; TG, triglycerides; EF, ejection fraction. | | | | |
| ^1^Categorical variables are expressed as count (percentage). Continuous variables are expressed as mean (standard deviation) if normally distributed, or median [interquartile range] if non-normally distributed. | | | | |

**Supplementary Table 4**. Baseline characteristics of patients stratified by DBP trajectory classes ^1^

| **Characteristics** | **Class 1:**  **Stable (n=456)** | **Class 2: High-decreasing-increasing (n=119)** | **Class 3: Low-increasing (n=161)** | ***P* value** |
| --- | --- | --- | --- | --- |
| **Demographics & Lifestyle** |  |  |  |  |
| Age, mean (SD) | 61.41 (10.06) | 57.40 (11.02) | 62.24 (9.88) | <0.001 |
| Sex, n (%) |  |  |  | 0.305 |
| Male | 346 (75.9) | 98 (82.4) | 122 (75.8) |  |
| Female | 110 (24.1) | 21 (17.6) | 39 (24.2) |  |
| BMI, mean (SD) | 24.73 (3.11) | 25.75 (3.22) | 24.47 (2.94) | 0.001 |
| Smoking, n (%) |  |  |  | 0.099 |
| Never | 270 (59.2) | 65 (54.6) | 83 (51.6) |  |
| Current | 139 (30.5) | 40 (33.6) | 67 (41.6) |  |
| Former | 47 (10.3) | 14 (11.8) | 11 (6.8) |  |
| Drinking, n (%) |  |  |  | 0.052 |
| Never | 378 (82.9) | 94 (79.0) | 118 (73.3) |  |
| Current | 75 (16.4) | 25 (21.0) | 40 (24.8) |  |
| Former | 3 (0.7) | 0 (0.0) | 3 (1.9) |  |
| **Medical History** |  |  |  |  |
| DM, n (%) |  |  |  | 0.013 |
| No | 311 (68.2) | 86 (72.3) | 92 (57.1) |  |
| Yes | 145 (31.8) | 33 (27.7) | 69 (42.9) |  |
| Hyperlipidemia, n (%) |  |  |  | 0.500 |
| No | 367 (80.5) | 91 (76.5) | 132 (82.0) |  |
| Yes | 89 (19.5) | 28 (23.5) | 29 (18.0) |  |
| Hyperuricemia, n (%) |  |  |  | 0.763 |
| No | 449 (98.5) | 116 (97.5) | 158 (98.1) |  |
| Yes | 7 (1.5) | 3 (2.5) | 3 (1.9) |  |
| Stroke, n (%) |  |  |  | 0.875 |
| No | 387 (84.9) | 103 (86.6) | 136 (84.5) |  |
| Yes | 69 (15.1) | 16 (13.4) | 25 (15.5) |  |
| HF, n (%) |  |  |  | 0.010 |
| No | 426 (93.4) | 110 (92.4) | 138 (85.7) |  |
| Yes | 30 (6.6) | 9 (7.6) | 23 (14.3) |  |
| NYHA functional class, n (%) |  |  |  | 0.906 |
| Normal/0 | 108 (23.7) | 26 (21.8) | 36 (22.4) |  |
| Class I | 48 (10.5) | 14 (11.8) | 22 (13.7) |  |
| Class II | 267 (58.6) | 71 (59.7) | 91 (56.5) |  |
| Class III | 30 (6.6) | 7 (5.9) | 9 (5.6) |  |
| Class IV | 3 (0.7) | 1 (0.8) | 3 (1.9) |  |
| Anemia, n (%) |  |  |  | 0.649 |
| No | 449 (98.5) | 116 (97.5) | 157 (97.5) |  |
| Yes | 7 (1.5) | 3 (2.5) | 4 (2.5) |  |
| Arrhythmia, n (%) |  |  |  | 0.217 |
| No | 404 (88.6) | 110 (92.4) | 138 (85.7) |  |
| Yes | 52 (11.4) | 9 (7.6) | 23 (14.3) |  |
| **Angiography & Interventions** |  |  |  |  |
| Left main disease, n (%) |  |  |  | 0.175 |
| No | 399 (87.5) | 105 (88.2) | 132 (82.0) |  |
| Yes | 57 (12.5) | 14 (11.8) | 29 (18.0) |  |
| LAD lesion, n (%) |  |  |  | 0.811 |
| No | 68 (14.9) | 16 (13.4) | 21 (13.0) |  |
| Yes | 388 (85.1) | 103 (86.6) | 140 (87.0) |  |
| LCX lesion, n (%) |  |  |  | 0.961 |
| No | 129 (28.3) | 35 (29.4) | 45 (28.0) |  |
| Yes | 327 (71.7) | 84 (70.6) | 116 (72.0) |  |
| RCA lesion, n (%) |  |  |  | 0.392 |
| No | 139 (30.5) | 44 (37.0) | 50 (31.1) |  |
| Yes | 317 (69.5) | 75 (63.0) | 111 (68.9) |  |
| Multivessel disease, n (%) |  |  |  | 0.552 |
| 0-vessel | 19 (4.2) | 5 (4.2) | 9 (5.6) |  |
| 1-vessel | 79 (17.3) | 23 (19.3) | 19 (11.8) |  |
| 2-vessel | 121 (26.5) | 34 (28.6) | 51 (31.7) |  |
| 3-vessel | 237 (52.0) | 57 (47.9) | 82 (50.9) |  |
| Stents in LM, mean (SD) | 0.11 (0.34) | 0.08 (0.30) | 0.16 (0.43) | 0.176 |
| Stents in LAD, mean (SD) | 0.85 (0.95) | 0.84 (1.03) | 0.86 (0.96) | 0.979 |
| Stents in LCX, mean (SD) | 0.23 (0.54) | 0.26 (0.56) | 0.29 (0.63) | 0.445 |
| Stents in RCA, mean (SD) | 0.51 (0.87) | 0.44 (0.92) | 0.37 (0.77) | 0.168 |
| Total number of stents, mean (SD) | 1.70 (1.00) | 1.61 (1.04) | 1.68 (1.05) | 0.699 |
| **Medications** |  |  |  |  |
| β-blockers, n (%) |  |  |  | 0.274 |
| No | 140 (30.7) | 30 (25.2) | 55 (34.2) |  |
| Yes | 316 (69.3) | 89 (74.8) | 106 (65.8) |  |
| ACEI/ARB, n (%) |  |  |  | 0.005 |
| No | 374 (82.0) | 87 (73.1) | 142 (88.2) |  |
| Yes | 82 (18.0) | 32 (26.9) | 19 (11.8) |  |
| Statin, n (%) |  |  |  | 0.285 |
| No | 28 (6.1) | 3 (2.5) | 8 (5.0) |  |
| Yes | 428 (93.9) | 116 (97.5) | 153 (95.0) |  |
| P2Y12 inhibitors, n (%) |  |  |  | 0.161 |
| No | 5 (1.1) | 0 (0.0) | 4 (2.5) |  |
| Yes | 451 (98.9) | 119 (100.0) | 157 (97.5) |  |
| Aspirin, n (%) |  |  |  | 0.018 |
| No | 30 (6.6) | 4 (3.4) | 19 (11.8) |  |
| Yes | 426 (93.4) | 115 (96.6) | 142 (88.2) |  |
| **Laboratory Results & Echo** |  |  |  |  |
| WBC, 10⁹/L (median [IQR]) | 7.03 [5.38, 8.40] | 7.29 [5.93, 8.96] | 7.03 [5.44, 9.13] | 0.160 |
| Hemoglobin, g/L (mean ± SD) | 138.08 (19.92) | 142.24 (17.47) | 135.53 (22.40) | 0.023 |
| Platelets, 10⁹/L (mean ± SD) | 203.94 (60.51) | 215.40 (67.21) | 207.43 (68.31) | 0.210 |
| HbA1c, % (mean ± SD) | 6.62 (1.39) | 6.41 (1.30) | 6.74 (1.57) | 0.156 |
| FBG, mmol/L (mean ± SD) | 6.34 (2.36) | 6.08 (1.94) | 6.37 (2.65) | 0.525 |
| Potassium, mmol/L (mean ± SD) | 3.97 (0.37) | 3.91 (0.34) | 4.00 (0.41) | 0.146 |
| Creatinine, μmol/L (median [IQR]) | 72.0 [60.0, 99.3] | 78.0 [66.0, 102.9] | 75.0 [61.0, 122.0] | 0.148 |
| HDL-C, mmol/L (mean ± SD) | 1.08 (0.39) | 1.04 (0.35) | 1.02 (0.37) | 0.256 |
| LDL-C, mmol/L (mean ± SD) | 2.29 (1.17) | 2.59 (1.20) | 2.28 (1.21) | 0.042 |
| Triglycerides, mmol/L (median [IQR]) | 3.82 [2.95, 4.60] | 3.94 [3.26, 4.91] | 3.53 [2.88, 4.47] | 0.014 |
| EF, mean (SD) | 58.80 (12.20) | 57.32 (12.76) | 56.12 (11.57) | 0.047 |
| Abbreviations: DBP, diastolic blood pressure; BMI, body mass index; DM, diabetes mellitus; HF, heart failure; NYHA, New York Heart Association; LM, left main; LAD, left anterior descending; LCX, left circumflex; RCA, right coronary artery; ACEI/ARB, angiotensin-converting enzyme inhibitors/angiotensin receptor blockers; WBC, white blood cell; Hb, hemoglobin; PLT, platelet; HbA1c, glycated hemoglobin; FBG, fasting blood glucose; K, potassium; Cr, creatinine; HDL-C, high-density lipoprotein cholesterol; LDL-C, low-density lipoprotein cholesterol; TG, triglycerides; EF, ejection fraction. | | | | |
| ^1^Categorical variables are expressed as count (percentage). Continuous variables are expressed as mean (standard deviation) if normally distributed, or median [interquartile range] if non-normally distributed. | | | | |

**Supplementary Table 5**. Baseline characteristics of patients stratified by RHR trajectory classes ^1^

| **Characteristics** | **Class 1: Fluctuating (n=192)** | **Class 2: Stable (n=544)** | **P value** |
| --- | --- | --- | --- |
| **Demographics & Lifestyle** |  |  |  |
| Age, mean (SD) | 60.49 (10.88) | 61.10 (10.08) | 0.477 |
| Sex, n (%) |  |  | 0.043 |
| Male | 137 (71.4) | 429 (78.9) |  |
| Female | 55 (28.6) | 115 (21.1) |  |
| BMI, mean (SD) | 24.42 (3.34) | 24.99 (3.03) | 0.030 |
| Smoking, n (%) |  |  | 0.395 |
| Never | 110 (57.3) | 308 (56.6) |  |
| Current | 59 (30.7) | 187 (34.4) |  |
| Former | 23 (12.0) | 49 (9.0) |  |
| Drinking, n (%) |  |  | 0.867 |
| Never | 154 (80.2) | 436 (80.1) |  |
| Current | 37 (19.3) | 103 (18.9) |  |
| Former | 1 (0.5) | 5 (0.9) |  |
| **Medical History** |  |  |  |
| DM, n (%) |  |  | 0.731 |
| No | 130 (67.7) | 359 (66.0) |  |
| Yes | 62 (32.3) | 185 (34.0) |  |
| Hyperlipidemia, n (%) |  |  | 1.000 |
| No | 154 (80.2) | 436 (80.1) |  |
| Yes | 38 (19.8) | 108 (19.9) |  |
| Hyperuricemia, n (%) |  |  | 0.945 |
| No | 188 (97.9) | 535 (98.3) |  |
| Yes | 4 (2.1) | 9 (1.7) |  |
| Stroke, n (%) |  |  | 0.850 |
| No | 162 (84.4) | 464 (85.3) |  |
| Yes | 30 (15.6) | 80 (14.7) |  |
| HF, n (%) |  |  | <0.001 |
| No | 162 (84.4) | 512 (94.1) |  |
| Yes | 30 (15.6) | 32 (5.9) |  |
| NYHA functional class, n (%) |  |  | 0.095 |
| Normal/0 | 46 (24.0) | 124 (22.8) |  |
| Class I | 27 (14.1) | 57 (10.5) |  |
| Class II | 100 (52.1) | 329 (60.5) |  |
| Class III | 15 (7.8) | 31 (5.7) |  |
| Class IV | 4 (2.1) | 3 (0.6) |  |
| Anemia, n (%) |  |  | 0.256 |
| No | 186 (96.9) | 536 (98.5) |  |
| Yes | 6 (3.1) | 8 (1.5) |  |
| Arrhythmia, n (%) |  |  | 0.495 |
| No | 167 (87.0) | 485 (89.2) |  |
| Yes | 25 (13.0) | 59 (10.8) |  |
| **Angiography & Interventions** |  |  |  |
| Left main disease, n (%) |  |  | 0.403 |
| No | 162 (84.4) | 474 (87.1) |  |
| Yes | 30 (15.6) | 70 (12.9) |  |
| LAD lesion, n (%) |  |  | 0.098 |
| No | 20 (10.4) | 85 (15.6) |  |
| Yes | 172 (89.6) | 459 (84.4) |  |
| LCX lesion, n (%) |  |  | 0.849 |
| No | 53 (27.6) | 156 (28.7) |  |
| Yes | 139 (72.4) | 388 (71.3) |  |
| RCA lesion, n (%) |  |  | 0.554 |
| No | 57 (29.7) | 176 (32.4) |  |
| Yes | 135 (70.3) | 368 (67.6) |  |
| Multivessel disease, n (%) |  |  | 0.288 |
| 0-vessel | 6 (3.1) | 27 (5.0) |  |
| 1-vessel | 33 (17.2) | 88 (16.2) |  |
| 2-vessel | 46 (24.0) | 160 (29.4) |  |
| 3-vessel | 107 (55.7) | 269 (49.4) |  |
| Stents in LM, mean (SD) | 0.15 (0.42) | 0.11 (0.33) | 0.192 |
| Stents in LAD, mean (SD) | 0.87 (0.96) | 0.84 (0.97) | 0.748 |
| Stents in LCX, mean (SD) | 0.27 (0.57) | 0.24 (0.56) | 0.598 |
| Stents in RCA, mean (SD) | 0.46 (0.87) | 0.47 (0.85) | 0.865 |
| Total number of stents, mean (SD) | 1.74 (0.98) | 1.66 (1.03) | 0.362 |
| **Medications** |  |  |  |
| β-blockers, n (%) |  |  | <0.001 |
| No | 34 (17.7) | 191 (35.1) |  |
| Yes | 158 (82.3) | 353 (64.9) |  |
| ACEI/ARB, n (%) |  |  | 0.176 |
| No | 164 (85.4) | 439 (80.7) |  |
| Yes | 28 (14.6) | 105 (19.3) |  |
| Statin, n (%) |  |  | 0.619 |
| No | 12 (6.2) | 27 (5.0) |  |
| Yes | 180 (93.8) | 517 (95.0) |  |
| P2Y12 inhibitors, n (%) |  |  | 0.907 |
| No | 3 (1.6) | 6 (1.1) |  |
| Yes | 189 (98.4) | 538 (98.9) |  |
| Aspirin, n (%) |  |  | 0.667 |
| No | 12 (6.2) | 41 (7.5) |  |
| Yes | 180 (93.8) | 503 (92.5) |  |
| **Laboratory Results & Echo** |  |  |  |
| WBC, 10⁹/L (median [IQR]) | 7.43 [5.69, 9.66] | 6.99 [5.34, 8.48] | 0.005 |
| Hemoglobin, g/L (mean ± SD) | 138.42 (23.32) | 138.12 (19.00) | 0.857 |
| Platelets, 10⁹/L (mean ± SD) | 219.43 (70.91) | 202.01 (60.00) | 0.001 |
| HbA1c, % (mean ± SD) | 6.59 (1.42) | 6.62 (1.42) | 0.827 |
| FBG, mmol/L (mean ± SD) | 6.33 (2.23) | 6.30 (2.41) | 0.882 |
| Potassium, mmol/L (mean ± SD) | 3.99 (0.37) | 3.96 (0.38) | 0.346 |
| Creatinine, μmol/L (median [IQR]) | 78.5 [62.0, 122.3] | 72.0 [60.0, 103.0] | 0.172 |
| HDL-C, mmol/L (mean ± SD) | 1.09 (0.37) | 1.05 (0.38) | 0.125 |
| LDL-C, mmol/L (mean ± SD) | 2.41 (1.02) | 2.31 (1.24) | 0.292 |
| Triglycerides, mmol/L (median [IQR]) | 3.95 [3.28, 4.72] | 3.63 [2.90, 4.56] | 0.004 |
| EF, mean (SD) | 57.17 (13.10) | 58.26 (11.86) | 0.290 |
| Abbreviations: RHR, resting heart rate; BMI, body mass index; DM, diabetes mellitus; HF, heart failure; NYHA, New York Heart Association; LM, left main; LAD, left anterior descending; LCX, left circumflex; RCA, right coronary artery; ACEI/ARB, angiotensin-converting enzyme inhibitors/angiotensin receptor blockers; WBC, white blood cell; Hb, hemoglobin; PLT, platelet; HbA1c, glycated hemoglobin; FBG, fasting blood glucose; K, potassium; Cr, creatinine; HDL-C, high-density lipoprotein cholesterol; LDL-C, low-density lipoprotein cholesterol; TG, triglycerides; EF, ejection fraction. | | | |
| ^1^Categorical variables are expressed as count (percentage). Continuous variables are expressed as mean (standard deviation) if normally distributed, or median [interquartile range] if non-normally distributed. | | | |

**Supplementary Table 6** Baseline Characteristics of Included vs. Excluded Patients

| **Characteristics** | **Included Group (n=736)** | **Excluded Group (n=204)** | ***P* value** |
| --- | --- | --- | --- |
| **Demographics & Lifestyle** |  |  |  |
| Age, mean (SD) | 60.94 (10.21) | 61.12 (10.45) | 0.812 |
| Sex, n (%) |  |  | 0.965 |
| Male | 566 (76.9) | 171 (77.0) |  |
| Female | 170 (23.1) | 51 (23.0) |  |
| BMI, mean (SD) | 24.91 (3.12) | 24.85 (3.20) | 0.795 |
| Smoking, n (%) |  |  | 0.884 |
| Never | 418 (56.8) | 125 (56.3) |  |
| Current | 246 (33.4) | 75 (33.8) |  |
| Former | 72 (9.8) | 22 (9.9) |  |
| Drinking, n (%) |  |  | 0.941 |
| Never | 590 (80.2) | 179 (80.6) |  |
| Current | 140 (19.0) | 41 (18.5) |  |
| Former | 6 (0.8) | 2 (0.9) |  |
| **Clinical Presentation, n (%)** |  |  | 0.724 |
| ACS | 565 (76.8) | 168 (75.7) |  |
| CCS | 171 (23.2) | 54 (24.3) |  |
| **Medical History** |  |  |  |
| DM, n (%) |  |  | 0.841 |
| No | 489 (66.4) | 149 (67.1) |  |
| Yes | 247 (33.6) | 73 (32.9) |  |
| Hyperlipidemia, n (%) |  |  | 0.816 |
| No | 590 (80.2) | 177 (79.7) |  |
| Yes | 146 (19.8) | 45 (20.3) |  |
| Hyperuricemia, n (%) |  |  | >0.99 |
| No | 723 (98.2) | 218 (98.2) |  |
| Yes | 13 (1.8) | 4 (1.8) |  |
| Stroke, n (%) |  |  | 0.852 |
| No | 626 (85.1) | 188 (84.7) |  |
| Yes | 110 (14.9) | 34 (15.3) |  |
| HF, n (%) |  |  | 0.725 |
| No | 674 (91.6) | 205 (92.3) |  |
| Yes | 62 (8.4) | 17 (7.7) |  |
| NYHA functional class, n (%) |  |  | 0.912 |
| Normal/0 | 170 (23.1) | 50 (22.5) |  |
| Class I | 84 (11.4) | 28 (12.6) |  |
| Class II | 429 (58.3) | 131 (59.0) |  |
| Class III | 46 (6.3) | 11 (5.0) |  |
| Class IV | 7 (1.0) | 2 (0.9) |  |
| Anemia, n (%) |  |  | 0.785 |
| No | 722 (98.1) | 218 (98.2) |  |
| Yes | 14 (1.9) | 4 (1.8) |  |
| Arrhythmia, n (%) |  |  | 0.887 |
| No | 652 (88.6) | 197 (88.7) |  |
| Yes | 84 (11.4) | 25 (11.3) |  |
| **Angiography & Interventions** |  |  |  |
| Left main disease, n (%) |  |  | 0.933 |
| No | 636 (86.4) | 192 (86.5) |  |
| Yes | 100 (13.6) | 30 (13.5) |  |
| LAD lesion, n (%) |  |  | 0.864 |
| No | 105 (14.3) | 31 (14.0) |  |
| Yes | 631 (85.7) | 191 (86.0) |  |
| LCX lesion, n (%) |  |  | 0.896 |
| No | 209 (28.4) | 64 (28.8) |  |
| Yes | 527 (71.6) | 158 (71.2) |  |
| RCA lesion, n (%) |  |  | 0.812 |
| No | 233 (31.7) | 69 (31.1) |  |
| Yes | 503 (68.3) | 153 (68.9) |  |
| Multivessel disease, n (%) |  |  | 0.923 |
| 0-vessel | 33 (4.5) | 11 (5.0) |  |
| 1-vessel | 121 (16.4) | 35 (15.8) |  |
| 2-vessel | 206 (28.0) | 59 (26.6) |  |
| 3-vessel | 376 (51.1) | 117 (52.7) |  |
| Stents in LM, mean (SD) | 0.11 (0.35) | 0.10 (0.33) | 0.704 |
| Stents in LAD, mean (SD) | 0.85 (0.97) | 0.82 (0.95) | 0.685 |
| Stents in LCX, mean (SD) | 0.25 (0.56) | 0.26 (0.58) | 0.821 |
| Stents in RCA, mean (SD) | 0.47 (0.86) | 0.45 (0.83) | 0.762 |
| Total number of stents, mean (SD) | 1.68 (1.02) | 1.65 (1.00) | 0.698 |
| **Medications** |  |  |  |
| β-blockers, n (%) |  |  | 0.801 |
| No | 225 (30.6) | 66 (29.7) |  |
| Yes | 511 (69.4) | 156 (70.3) |  |
| ACEI/ARB, n (%) |  |  | 0.785 |
| No | 603 (81.9) | 182 (82.0) |  |
| Yes | 133 (18.1) | 40 (18.0) |  |
| Statin, n (%) |  |  | 0.793 |
| No | 39 (5.3) | 12 (5.4) |  |
| Yes | 697 (94.7) | 210 (94.6) |  |
| P2Y12 inhibitors, n (%) |  |  | 0.672 |
| No | 9 (1.2) | 3 (1.4) |  |
| Yes | 727 (98.8) | 219 (98.6) |  |
| Aspirin, n (%) |  |  | 0.963 |
| No | 53 (7.2) | 16 (7.2) |  |
| Yes | 683 (92.8) | 206 (92.8) |  |
| **Laboratory Results & Echo** |  |  |  |
| WBC, 10⁹/L (median [IQR]) | 7.05 [5.30, 8.81] | 7.10 [5.40, 8.90] | 0.654 |
| Hemoglobin, g/L (mean ± SD) | 138.19 (20.35) | 137.50 (21.10) | 0.662 |
| Platelets, 10⁹/L (mean ± SD) | 206.56 (63.85) | 205.10 (62.50) | 0.760 |
| HbA1c, % (mean ± SD) | 6.61 (1.41) | 6.64 (1.45) | 0.783 |
| FBG, mmol/L (mean ± SD) | 6.30 (2.36) | 6.35 (2.42) | 0.785 |
| Potassium, mmol/L (mean ± SD) | 3.97 (0.38) | 3.95 (0.36) | 0.495 |
| Creatinine, μmol/L (median [IQR]) | 74.0 [61.0, 108.0] | 73.0 [62.0, 105.0] | 0.811 |
| HDL-C, mmol/L (mean ± SD) | 1.06 (0.38) | 1.08 (0.36) | 0.492 |
| LDL-C, mmol/L (mean ± SD) | 2.34 (1.18) | 2.32 (1.15) | 0.820 |
| Triglycerides, mmol/L (median [IQR]) | 3.75 [3.01, 4.60] | 3.70 [2.95, 4.55] | 0.684 |
| EF, mean (SD) | 57.97 (12.28) | 58.10 (12.15) | 0.890 |
| Abbreviations: BMI, body mass index; ACS, acute coronary syndrome; CCS, chronic coronary syndrome; DM, diabetes mellitus; HF, heart failure; NYHA, New York Heart Association; LM, left main; LAD, left anterior descending; LCX, left circumflex; RCA, right coronary artery; ACEI, angiotensin-converting enzyme inhibitor; ARB, angiotensin receptor blocker; WBC, white blood cell; Hb, hemoglobin; PLT, platelet; HbA1c, glycated hemoglobin; FBG, fasting blood glucose; K, potassium; Cr, creatinine; HDL-C, high-density lipoprotein cholesterol; LDL-C, low-density lipoprotein cholesterol; TG, triglycerides; EF, ejection fraction. | | | |
| Continuous variables are presented as mean (standard deviation, SD) or median [interquartile range, IQR] and compared using Student’s t-test or Mann-Whitney U test, as appropriate. Categorical variables are presented as frequencies (percentages) and compared using the Chi-square test or Fisher’s exact test. | | | |

**Table 1**. Baseline characteristics of study participants stratified by BP and RHR control status ^1^

| **Characteristics** | **Unweighted Cohort (N = 736)** | | | | | | **IPTW-Weighted Cohort (Pseudo-population)** | | | | | |
| --- | --- | --- | --- | --- | --- | --- | --- | --- | --- | --- | --- | --- |
|  | **Double Stable (n=329)** | **BP-Fluctuating (n=215)** | **RHR-Fluctuating (n=108)** | **Double Fluctuating (n=84)** | **P value** | **SMD** | **Double Stable** | **BP-Fluctuating** | **RHR-Fluctuating** | **Double Fluctuating** | **P value** | **SMD** |
| **Demographics & Lifestyle** |  |  |  |  |  |  |  |  |  |  |  |  |
| Age, years (mean ± SD) | 61.36 ± 10.26 | 60.71 ± 9.81 | 60.95 ± 10.43 | 59.89 ± 11.48 | 0.674 | 0.073 | 61.03 ± 10.58 | 60.78 ± 9.85 | 60.91 ± 10.01 | 60.76 ± 9.91 | 0.993 | 0.016 |
| Sex, n (%) |  |  |  |  | 0.190 | 0.128 |  |  |  |  | 0.983 | 0.029 |
| Male | 257 (78.1) | 172 (80.0) | 77 (71.3) | 60 (71.4) |  |  | 254.7 (77.3) | 162.3 (76.6) | 84.2 (78.6) | 66.3 (76.4) |  |  |
| Female | 72 (21.9) | 43 (20.0) | 31 (28.7) | 24 (28.6) |  |  | 74.9 (22.7) | 49.6 (23.4) | 23.0 (21.4) | 20.4 (23.6) |  |  |
| BMI, kg/m² (mean ± SD) | 25.05 ± 3.07 | 24.77 ± 2.88 | 24.54 ± 3.19 | 24.27 ± 3.40 | 0.144 | 0.135 | 24.79 ± 3.17 | 24.85 ± 2.92 | 24.55 ± 3.14 | 25.22 ± 3.61 | 0.797 | 0.105 |
| Smoking, n (%) |  |  |  |  | 0.827 | 0.103 |  |  |  |  | 0.996 | 0.055 |
| Never | 191 (58.1) | 117 (54.4) | 61 (56.5) | 49 (58.3) |  |  | 185.5 (56.3) | 120.6 (56.9) | 59.7 (55.7) | 46.5 (53.6) |  |  |
| Current | 109 (33.1) | 78 (36.3) | 33 (30.6) | 26 (31.0) |  |  | 108.2 (32.8) | 70.2 (33.1) | 36.3 (33.9) | 32.2 (37.1) |  |  |
| Former | 29 (8.8) | 20 (9.3) | 14 (13.0) | 9 (10.7) |  |  | 35.9 (10.9) | 21.2 (10.0) | 11.2 (10.4) | 8.0 (9.3) |  |  |
| Drinking, n (%) |  |  |  |  | 0.868 | 0.127 |  |  |  |  | 0.662 | 0.096 |
| Never | 265 (80.5) | 171 (79.5) | 84 (77.8) | 70 (83.3) |  |  | 266.4 (80.8) | 168.6 (79.6) | 83.0 (77.4) | 64.5 (74.4) |  |  |
| Current | 61 (18.5) | 42 (19.5) | 24 (22.2) | 13 (15.5) |  |  | 60.2 (18.3) | 40.4 (19.1) | 24.2 (22.6) | 19.0 (21.9) |  |  |
| Former | 3 (0.9) | 2 (0.9) | 0 (0.0) | 1 (1.2) |  |  | 3.1 (0.9) | 2.9 (1.4) | 0.0 (0.0) | 3.2 (3.6) |  |  |
| **Clinical Presentation & History** |  |  |  |  |  |  |  |  |  |  |  |  |
| Clinical Presentation, n (%) |  |  |  |  | 0.002 | 0.242 |  |  |  |  | 0.793 | 0.076 |
| ACS | 257 (78.1) | 175 (81.4) | 82 (75.9) | 51 (60.7) |  |  | 255.6 (77.5) | 165.9 (78.3) | 87.6 (81.7) | 65.7 (75.8) |  |  |
| CCS | 72 (21.9) | 40 (18.6) | 26 (24.1) | 33 (39.3) |  |  | 74.0 (22.5) | 46.0 (21.7) | 19.6 (18.3) | 21.0 (24.2) |  |  |
| Diabetes mellitus, n (%) | 117 (35.6) | 68 (31.6) | 33 (30.6) | 29 (34.5) | 0.700 | 0.064 | 114.8 (34.8) | 72.1 (34.0) | 36.6 (34.2) | 27.8 (32.1) | 0.980 | 0.030 |
| Hyperlipidemia, n (%) | 65 (19.8) | 43 (20.0) | 24 (22.2) | 14 (16.7) | 0.820 | 0.071 | 71.1 (21.6) | 44.8 (21.1) | 23.0 (21.4) | 12.6 (14.5) | 0.615 | 0.094 |
| Hyperuricemia, n (%) | 6 (1.8) | 3 (1.4) | 3 (2.8) | 1 (1.2) | 0.808 | 0.063 | 6.9 (2.1) | 3.1 (1.4) | 2.1 (1.9) | 6.0 (6.9) | 0.267 | 0.092 |
| Stroke, n (%) | 52 (15.8) | 28 (13.0) | 14 (13.0) | 16 (19.0) | 0.520 | 0.096 | 52.5 (15.9) | 30.9 (14.6) | 14.5 (13.5) | 13.6 (15.7) | 0.955 | 0.039 |
| Heart failure, n (%) | 15 (4.6) | 17 (7.9) | 16 (14.8) | 14 (16.7) | <0.001 | 0.238 | 28.8 (8.7) | 16.4 (7.7) | 8.2 (7.7) | 5.4 (6.2) | 0.851 | 0.049 |
| NYHA functional class, n (%) |  |  |  |  | 0.155 | 0.256 |  |  |  |  | 0.599 | 0.099 |
| Normal/0 | 79 (24.0) | 45 (20.9) | 25 (23.1) | 21 (25.0) |  |  | 80.3 (24.3) | 47.2 (22.3) | 22.3 (20.8) | 18.9 (21.8) |  |  |
| Class I | 33 (10.0) | 24 (11.2) | 16 (14.8) | 11 (13.1) |  |  | 33.2 (10.1) | 22.8 (10.8) | 19.2 (17.9) | 13.2 (15.2) |  |  |
| Class II | 202 (61.4) | 127 (59.1) | 60 (55.6) | 40 (47.6) |  |  | 199.2 (60.4) | 123.0 (58.0) | 60.6 (56.5) | 46.0 (53.1) |  |  |
| Class III | 14 (4.3) | 17 (7.9) | 5 (4.6) | 10 (11.9) |  |  | 13.9 (4.2) | 16.3 (7.7) | 3.5 (3.2) | 8.0 (9.3) |  |  |
| Class IV | 1 (0.3) | 2 (0.9) | 2 (1.9) | 2 (2.4) |  |  | 3.0 (0.9) | 2.7 (1.3) | 1.7 (1.6) | 0.6 (0.7) |  |  |
| Anemia, n (%) | 5 (1.5) | 3 (1.4) | 3 (2.8) | 3 (3.6) | 0.522 | 0.085 | 5.3 (1.6) | 2.9 (1.4) | 3.4 (3.1) | 2.1 (2.5) | 0.684 | 0.071 |
| Arrhythmia, n (%) | 33 (10.0) | 26 (12.1) | 14 (13.0) | 11 (13.1) | 0.750 | 0.052 | 32.3 (9.8) | 26.1 (12.3) | 10.6 (9.9) | 7.9 (9.1) | 0.791 | 0.053 |
| **Angiography & Interventions** |  |  |  |  |  |  |  |  |  |  |  |  |
| Left main disease, n (%) | 42 (12.8) | 28 (13.0) | 15 (13.9) | 15 (17.9) | 0.668 | 0.075 | 44.8 (13.6) | 31.5 (14.9) | 16.7 (15.6) | 10.2 (11.7) | 0.892 | 0.063 |
| LAD lesion, n (%) | 284 (86.3) | 175 (81.4) | 94 (87.0) | 78 (92.9) | 0.071 | 0.178 | 286.3 (86.9) | 172.0 (81.1) | 93.8 (87.5) | 81.0 (93.4) | 0.062 | 0.092 |
| LCX lesion, n (%) | 234 (71.1) | 154 (71.6) | 73 (67.6) | 66 (78.6) | 0.408 | 0.126 | 235.7 (71.5) | 148.8 (70.2) | 75.4 (70.3) | 60.9 (70.2) | 0.989 | 0.015 |
| RCA lesion, n (%) | 218 (66.3) | 150 (69.8) | 81 (75.0) | 54 (64.3) | 0.294 | 0.130 | 219.9 (66.7) | 149.1 (70.3) | 80.1 (74.7) | 50.7 (58.5) | 0.214 | 0.087 |
| Multivessel disease, n (%) |  |  |  |  | 0.235 | 0.198 |  |  |  |  | 0.575 | 0.096 |
| 0-vessel | 11 (3.3) | 16 (7.4) | 3 (2.8) | 3 (3.6) |  |  | 10.3 (3.1) | 15.6 (7.4) | 3.9 (3.7) | 4.3 (4.9) |  |  |
| 1-vessel | 59 (17.9) | 29 (13.5) | 21 (19.4) | 12 (14.3) |  |  | 60.4 (18.3) | 29.8 (14.0) | 16.8 (15.6) | 18.5 (21.3) |  |  |
| 2-vessel | 100 (30.4) | 60 (27.9) | 25 (23.1) | 21 (25.0) |  |  | 95.4 (28.9) | 59.4 (28.0) | 27.0 (25.2) | 17.8 (20.6) |  |  |
| 3-vessel | 159 (48.3) | 110 (51.2) | 59 (54.6) | 48 (57.1) |  |  | 163.6 (49.6) | 107.1 (50.5) | 59.5 (55.5) | 46.2 (53.2) |  |  |
| Stents in LM, mean ± SD | 0.13 ± 0.37 | 0.07 ± 0.26 | 0.08 ± 0.31 | 0.23 ± 0.52 | 0.007 | 0.207 | 0.12 ± 0.36 | 0.10 ± 0.29 | 0.10 ± 0.35 | 0.11 ± 0.35 | 0.803 | 0.046 |
| Stents in LAD, mean ± SD | 0.80 ± 0.95 | 0.91 ± 0.98 | 0.94 ± 1.03 | 0.77 ± 0.87 | 0.342 | 0.108 | 0.81 ± 0.96 | 0.85 ± 0.95 | 0.89 ± 1.04 | 0.91 ± 0.85 | 0.811 | 0.061 |
| Stents in LCX, mean ± SD | 0.25 ± 0.57 | 0.23 ± 0.54 | 0.28 ± 0.59 | 0.25 ± 0.53 | 0.926 | 0.041 | 0.25 ± 0.58 | 0.21 ± 0.52 | 0.26 ± 0.57 | 0.25 ± 0.55 | 0.882 | 0.044 |
| Stents in RCA, mean ± SD | 0.46 ± 0.83 | 0.49 ± 0.89 | 0.46 ± 0.85 | 0.45 ± 0.91 | 0.979 | 0.021 | 0.49 ± 0.86 | 0.51 ± 0.89 | 0.47 ± 0.83 | 0.40 ± 0.85 | 0.861 | 0.065 |
| Total number of stents, mean ± SD | 1.63 ± 1.05 | 1.71 ± 1.00 | 1.77 ± 1.04 | 1.70 ± 0.92 | 0.630 | 0.068 | 1.66 ± 1.04 | 1.67 ± 0.98 | 1.72 ± 0.97 | 1.67 ± 0.93 | 0.977 | 0.028 |
| **Discharge Medications** |  |  |  |  |  |  |  |  |  |  |  |  |
| β-blockers, n (%) | 211 (64.1) | 142 (66.0) | 91 (84.3) | 67 (79.8) | <0.001 | 0.288 | 228.7 (69.4) | 145.1 (68.5) | 73.3 (68.3) | 58.1 (67.0) | 0.985 | 0.026 |
| ACEI/ARB, n (%) | 63 (19.1) | 42 (19.5) | 16 (14.8) | 12 (14.3) | 0.541 | 0.089 | 59.1 (17.9) | 37.8 (17.8) | 18.1 (16.9) | 18.7 (21.5) | 0.902 | 0.059 |
| Statin, n (%) | 313 (95.1) | 204 (94.9) | 103 (95.4) | 77 (91.7) | 0.620 | 0.077 | 315.3 (95.7) | 201.7 (95.2) | 103.7 (96.8) | 82.7 (95.4) | 0.927 | 0.043 |
| P2Y12 inhibitors, n (%) | 327 (99.4) | 211 (98.1) | 107 (99.1) | 82 (97.6) | 0.429 | 0.088 | 328.0 (99.5) | 207.9 (98.1) | 106.7 (99.6) | 86.2 (99.4) | 0.115 | 0.072 |
| Aspirin, n (%) | 303 (92.1) | 200 (93.0) | 103 (95.4) | 77 (91.7) | 0.685 | 0.081 | 304.6 (92.4) | 195.6 (92.3) | 103.9 (96.9) | 79.7 (92.0) | 0.486 | 0.090 |
| **Laboratory Results & Echo** |  |  |  |  |  |  |  |  |  |  |  |  |
| WBC, 10⁹/L (median [IQR]) | 6.91 [5.19, 8.32] | 7.03 [5.50, 8.86] | 7.08 [5.40, 9.17] | 7.53 [6.39, 9.89] | 0.006 | 0.234 | 7.03 [5.61, 8.52] | 7.03 [5.49, 8.93] | 6.99 [5.37, 9.19] | 7.23 [5.69, 8.68] | 0.997 | 0.027 |
| Hemoglobin, g/L (mean ± SD) | 138.90 ± 17.15 | 136.91 ± 21.52 | 139.96 ± 21.11 | 136.44 ± 25.88 | 0.435 | 0.097 | 138.91 ± 17.38 | 136.43 ± 20.95 | 140.63 ± 20.64 | 137.30 ± 19.33 | 0.354 | 0.102 |
| Platelets, 10⁹/L (mean ± SD) | 201.39 ± 59.27 | 202.96 ± 61.24 | 218.71 ± 72.11 | 220.36 ± 69.75 | 0.013 | 0.184 | 206.77 ± 61.89 | 206.85 ± 62.52 | 205.10 ± 65.46 | 207.04 ± 61.92 | 0.996 | 0.015 |
| HbA1c, % (mean ± SD) | 6.69 ± 1.42 | 6.52 ± 1.41 | 6.60 ± 1.48 | 6.58 ± 1.35 | 0.582 | 0.063 | 6.69 ± 1.44 | 6.58 ± 1.46 | 6.69 ± 1.41 | 6.43 ± 1.12 | 0.348 | 0.093 |
| FBG, mmol/L (mean ± SD) | 6.40 ± 2.50 | 6.14 ± 2.26 | 6.16 ± 1.80 | 6.53 ± 2.69 | 0.428 | 0.101 | 6.42 ± 2.51 | 6.26 ± 2.39 | 6.21 ± 1.69 | 6.41 ± 2.42 | 0.802 | 0.057 |
| Potassium, mmol/L (mean ± SD) | 3.96 ± 0.37 | 3.96 ± 0.40 | 3.94 ± 0.37 | 4.06 ± 0.36 | 0.132 | 0.162 | 3.98 ± 0.37 | 3.98 ± 0.40 | 3.92 ± 0.35 | 4.01 ± 0.32 | 0.444 | 0.102 |
| Creatinine, μmol/L (median [IQR]) | 72.0 [60.0, 100.0] | 74.0 [61.0, 107.0] | 80.0 [59.8, 116.0] | 75.0 [63.0, 126.0] | 0.407 | 0.120 | 73.0 [60.0, 103.0] | 74.1 [61.0, 111.3] | 80.0 [59.0, 115.6] | 75.6 [63.0, 123.7] | 0.790 | 0.011 |
| HDL-C, mmol/L (mean ± SD) | 1.05 ± 0.39 | 1.04 ± 0.37 | 1.11 ± 0.38 | 1.07 ± 0.34 | 0.408 | 0.107 | 1.05 ± 0.39 | 1.05 ± 0.37 | 1.11 ± 0.39 | 1.08 ± 0.33 | 0.720 | 0.094 |
| LDL-C, mmol/L (mean ± SD) | 2.32 ± 1.21 | 2.29 ± 1.30 | 2.43 ± 1.07 | 2.39 ± 0.98 | 0.741 | 0.072 | 2.33 ± 1.22 | 2.35 ± 1.30 | 2.45 ± 1.19 | 2.33 ± 0.97 | 0.938 | 0.053 |
| Triglycerides, mmol/L (median [IQR]) | 3.75 [2.94, 4.58] | 3.53 [2.83, 4.46] | 3.88 [3.24, 4.68] | 4.08 [3.33, 4.75] | 0.015 | 0.160 | 3.76 [2.94, 4.59] | 3.66 [2.90, 4.72] | 3.83 [3.17, 4.64] | 3.96 [3.10, 4.59] | 0.641 | 0.052 |
| LVEF, % (mean ± SD) | 58.22 ± 11.94 | 58.31 ± 11.75 | 57.28 ± 13.73 | 57.03 ± 12.32 | 0.766 | 0.064 | 57.87 ± 12.12 | 58.03 ± 12.02 | 58.98 ± 12.45 | 56.75 ± 12.72 | 0.789 | 0.092 |
| Abbreviations: IPTW, inverse probability of treatment weighting; SMD, standardized mean difference; BMI, body mass index; ACS, acute coronary syndrome; CCS, chronic coronary syndrome; NYHA, New York Heart Association; LM, left main; LAD, left anterior descending; LCX, left circumflex; RCA, right coronary artery; ACEI, angiotensin-converting enzyme inhibitor; ARB, angiotensin receptor blocker; WBC, white blood cell; HbA1c, glycated hemoglobin; FBG, fasting blood glucose; HDL-C, high-density lipoprotein cholesterol; LDL-C, low-density lipoprotein cholesterol; LVEF, left ventricular ejection fraction. | | | | | | | | | | | | |
| Normally distributed continuous variables are presented as mean ± standard deviation (SD), non-normally distributed continuous variables as median [interquartile range (IQR)], and categorical variables as count (percentage). For between-group comparisons in the unweighted cohort: one-way analysis of variance (ANOVA) was used for normally distributed continuous variables, the Kruskal-Wallis H test for non-normally distributed continuous variables, and Pearson’s Chi-square test or Fisher’s exact test for categorical variables, as appropriate. | | | | | | | | | | | | |
| Values in the inverse probability of treatment weighting (IPTW)-weighted cohort are pseudo-population estimates derived from generalized propensity scores, and thus frequencies are presented with decimal precision to ensure statistical accuracy. The IPTW model was constructed using a multinomial logistic regression to estimate the probability of each patient being assigned to one of the four joint BP-RHR trajectory groups. The model was pre-specified to include 17 clinically relevant baseline covariates: age, sex, body mass index (BMI), smoking status, drinking status, clinical presentation (acute coronary syndrome [ACS] vs. chronic coronary syndrome [CCS]), diabetes mellitus, heart failure, history of stroke, baseline left ventricular ejection fraction (LVEF), multivessel coronary artery disease, number of stents implanted in the left main (LM) coronary artery, prescription of β-blockers at discharge, prescription of angiotensin-converting enzyme inhibitors/angiotensin II receptor blockers (ACEI/ARB) at discharge, prescription of statins at discharge, baseline serum creatinine, and baseline low-density lipoprotein cholesterol (LDL-C). To reduce variance and potential instability caused by extreme weights, the calculated propensity weights were symmetrically truncated at the 1st and 99th percentiles. | | | | | | | | | | | | |
| Standardized mean differences (SMDs) were used to assess the balance of covariates across groups before and after weighting. An SMD < 0.10 indicates excellent covariate balance, and an SMD < 0.20 is considered clinically acceptable. After IPTW weighting, all 17 covariates achieved optimal balance (all SMDs < 0.11, with a maximum SMD of 0.105 for BMI), indicating sufficient balancing of baseline confounders between groups. Therefore, no additional covariates were adjusted for in the primary weighted Cox proportional hazards models. | | | | | | | | | | | | |

**Supplementary Table 8**. Actual SBP, DBP, and RHR values at baseline and follow-up visits stratified by joint trajectory classes

| **Parameter** | **Time Point** | **Double Stable(n=329)** | **BP-Fluctuating(n=215)** | **RHR-Fluctuating(n=108)** | **Double Fluctuating(n=84)** |
| --- | --- | --- | --- | --- | --- |
| **SBP, mmHg** |  |  |  |  |  |
|  | Baseline (Month 0) | 124.1 (12.2) | 129.5 (11.4) | 124.9 (25.2) | 129.3 (27.9) |
|  | Month 1 | 123.0 (10.5) | 121.0 (10.8) | 123.0 (11.3) | 125.7 (12.7) |
|  | Month 3 | 121.5 (10.1) | 119.7 (10.5) | 124.8 (12.9) | 123.0 (11.2) |
|  | Month 6 | 122.2 (10.9) | 120.6 (10.0) | 124.9 (11.8) | 123.5 (11.9) |
| **DBP, mmHg** |  |  |  |  |  |
|  | Baseline (Month 0) | 75.7 (10.4) | 82.0 (12.2) | 76.3 (12.7) | 81.9 (14.9) |
|  | Month 1 | 72.6 (7.2) | 72.3 (7.5) | 72.2 (8.0) | 75.1 (8.1) |
|  | Month 3 | 72.4 (7.2) | 73.4 (8.0) | 71.0 (7.1) | 75.3 (8.2) |
|  | Month 6 | 72.7 (7.3) | 73.4 (7.6) | 71.6 (7.2) | 76.0 (8.6) |
| **RHR, bpm** |  |  |  |  |  |
|  | Baseline (Month 0) | 71.7 (9.2) | 70.0 (9.3) | 91.8 (9.8) | 93.1 (9.7) |
|  | Month 1 | 71.2 (7.9) | 71.3 (8.0) | 72.0 (8.8) | 70.5 (6.8) |
|  | Month 3 | 70.7 (8.5) | 70.3 (7.9) | 68.6 (7.8) | 72.4 (6.6) |
|  | Month 6 | 70.2 (8.4) | 69.9 (7.7) | 68.5 (7.7) | 75.2 (7.0) |
| Abbreviations: SBP, systolic blood pressure; DBP, diastolic blood pressure; RHR, resting heart rate; bpm, beats per minute. | | | | | |
| Data are presented as mean (standard deviation). | | | | | |

**Supplementary Table 9**. Association of SBP, DBP, RHR, and Joint Trajectories with Risk of Cardiovascular Events^1^

| **Variable & Group** | **Crude HR (95% CI)** | ***P* value** | **Model 1 HR (95% CI)** | ***P* value** | **Model 2 HR (95% CI)** | ***P* value** | **Model 3 HR (95% CI)** | ***P* value** |
| --- | --- | --- | --- | --- | --- | --- | --- | --- |
| **SBP Trajectories** |  |  |  |  |  |  |  |  |
| Group 2 (Ref) | 1.00 (Reference) | - | 1.00 (Reference) | - | 1.00 (Reference) | - | 1.00 (Reference) | - |
| Group 1 | 2.16 (1.11-4.22) | 0.035 | 2.24 (1.09-4.63) | 0.031 | 2.18 (1.02-4.65) | 0.044 | 2.77 (1.10-6.99) | 0.032 |
| Group 3 | 1.75 (1.03-2.99) | 0.039 | 2.04 (1.13-3.68) | 0.017 | 2.11 (1.05-4.25) | 0.037 | 2.75 (1.10-6.87) | 0.029 |
| P for trend | - | 0.589 | - | 0.509 | - | 0.542 | - | 0.699 |
| **DBP Trajectories** |  |  |  |  |  |  |  |  |
| Group 1 (Ref) | 1.00 (Reference) | - | 1.00 (Reference) | - | 1.00 (Reference) | - | 1.00 (Reference) | - |
| Group 2 | 1.97 (0.91-4.25) | 0.084 | 1.68 (0.73-3.88) | 0.225 | 1.40 (0.56-3.49) | 0.472 | 1.34 (0.61-3.01) | 0.340 |
| Group 3 | 1.85 (0.93-3.65) | 0.078 | 2.10 (1.02-4.33) | 0.045 | 2.16 (0.90-5.21) | 0.085 | 2.57 (0.97-6.81) | 0.057 |
| P for trend | - | 0.140 | - | 0.033 | - | 0.086 | - | 0.030 |
| **RHR Trajectories** |  |  |  |  |  |  |  |  |
| Group 2 (Ref) | 1.00 (Reference) | - | 1.00 (Reference) | - | 1.00 (Reference) | - | 1.00 (Reference) | - |
| Group 1 | 2.15 (1.23-3.74) | 0.007 | 3.11 (1.78-5.44) | <0.001 | 3.20 (1.67-6.14) | 0.001 | 3.63 (1.94-6.80) | <0.001 |
| P for trend | - | - | - | - | - | - | - | - |
| **Joint Trajectories** |  |  |  |  |  |  |  |  |
| Group 1 (Ref) | 1.00 (Reference) | - | 1.00 (Reference) | - | 1.00 (Reference) | - | 1.00 (Reference) | - |
| Group 2 | 2.24 (1.24-4.04) | 0.008 | 2.63 (1.39-4.98) | 0.003 | 2.60 (1.49-4.53) | 0.002 | 3.02 (1.75-5.21) | 0.001 |
| Group 3 | 2.40 (1.12-5.17) | 0.025 | 3.38 (2.16-5.31) | <0.001 | 3.91 (2.49-6.15) | <0.001 | 4.41 (2.85-6.83) | <0.001 |
| Group 4 | 3.82 (1.65-8.87) | 0.002 | 4.07 (2.16-7.67) | 0.002 | 4.40 (2.68-7.25) | <0.001 | 4.64 (2.74-7.86) | <0.001 |
| P for trend | - | 0.001 | - | <0.001 | - | <0.001 | - |  |
| Abbreviations: RHR, resting heart rate; CI, confidence interval; SBP, systolic blood pressure; DBP, diastolic blood pressure. | | | | | | | | |
| Model 1: Adjusted for age and sex | | | | | | | | |
| Model 2: Adjusted for variables in Model 1 plus initial clinical presentation (acute coronary syndrome vs. chronic coronary syndrome), diabetes mellitus, and heart failure. | | | | | | | | |
| Model 3: Adjusted for variables in Model 2 plus number of stents in the left main (LM) coronary artery, use of β-blockers, ACEI/ARB, white blood cell (WBC) count, and triglycerides. | | | | | | | | |
| ^1^P for trend was calculated by entering the trajectory groups as a continuous variable in the Cox proportional hazards model. | | | | | | | | |

**Supplementary Table 10** Subgroup analyses for the association between the high-risk joint trajectory and the risk of clinical outcomes

| Subgroup | Events (n) / Total (N) | IPTW HR (95% CI)* | *P* value | *P* for interaction |
| --- | --- | --- | --- | --- |
| **Age** |  |  |  | 0.057 |
| < 65 years | 45 / 482 | 3.44 (1.42-8.34) | 0.005 |  |
| ≥ 65 years | 26 / 254 | 1.90 (1.06-3.42) | 0.047 |  |
| **Sex** |  |  |  | 0.721 |
| Male | 25 / 247 | 2.72 (1.22-6.07) | 0.012 |  |
| Female | 46 / 489 | 2.62 (1.18-5.85) | 0.018 |  |
| **Diabetes Mellitus (DM)** |  |  |  | 0.352 |
| Yes | 28 / 265 | 2.42 (1.03-5.68) | 0.038 |  |
| No | 43 / 471 | 2.87 (1.35-6.19) | 0.006 |  |
| **Smoking Status** |  |  |  | 0.551 |
| Never | 42 / 418 | 2.75 (1.28-5.92) | 0.009 |  |
| Former | 6 / 72 | 2.48 (0.92-6.65) | 0.072 |  |
| Current | 23 / 246 | 2.67 (1.12-6.38) | 0.028 |  |
| **Heart Failure (HF)** |  |  |  | 0.038 |
| Yes | 11 / 62 | 1.42 (0.63-3.22) | 0.360 |  |
| No | 60 / 674 | 2.96 (1.47-5.98) | 0.002 |  |
| **Clinical Presentation** |  |  |  | 0.617 |
| ACS | 55 / 565 | 2.78 (1.37-5.66) | 0.008 |  |
| CCS | 16 / 171 | 2.55 (1.08-6.01) | 0.042 |  |
| **β-blocker Use** |  |  |  | 0.473 |
| Yes | 46 / 511 | 2.59 (1.27-5.29) | 0.017 |  |
| No | 25 / 225 | 2.86 (1.29-6.35) | 0.013 |  |
| Abbreviations: IPTW, inverse probability of treatment weighting; HR, hazard ratio; CI, confidence interval; NACE, net adverse clinical events; BP, blood pressure; RHR, resting heart rate; ACS, acute coronary syndrome; CCS, chronic coronary syndrome; DM, diabetes mellitus; HF, heart failure. | | | | |
| *IPTW-adjusted HR (95% CI) was calculated for the high-risk Double Fluctuating joint BP-RHR trajectory group (Group 4) compared with the Double Stable reference group (Group 1) within each subgroup stratum. The IPTW model was consistent with the primary analysis, balancing all predefined baseline confounders across trajectory groups | | | | |

In the heart failure subgroup (n=62, events=11), the observed trend suggested a potential increased risk (HR≈1.42), but given the limited sample size, this estimate is statistically unstable and should be interpreted with great caution.

**Supplementary Table 11** Sensitivity analyses for the associations between blood pressure, RHR Trajectories, and clinical outcomes.

| **Variables & Groups** | **Sensitivity Analysis 1** | | **Sensitivity Analysis 2** | |
| --- | --- | --- | --- | --- |
|  | **IPTW HR (95% CI)** | **P value** | **IPTW HR (95% CI)** | **P value** |
| **SBP Trajectories** |  |  |  |  |
| Group 2 (Ref) | 1.00 (Reference) | — | 1.00 (Reference) | — |
| Group 1 | 2.30 (1.20-4.40) | 0.012 | 2.48 (1.31-4.70) | 0.007 |
| Group 3 | 1.72 (1.01-2.95) | 0.048 | 1.80 (1.04-3.12) | 0.038 |
| **DBP Trajectories** |  |  |  |  |
| Group 1 (Ref) | 1.00 (Reference) | — | 1.00 (Reference) | — |
| Group 2 | 1.08 (0.45-2.58) | 0.860 | 1.12 (0.48-2.65) | 0.790 |
| Group 3 | 1.02 (0.40-2.60) | 0.960 | 1.06 (0.42-2.68) | 0.890 |
| **RHR Trajectories** |  |  |  |  |
| Group 2 (Ref) | 1.00 (Reference) | — | 1.00 (Reference) | — |
| Group 1 | 1.48 (1.02-2.15) | 0.040 | 1.55 (1.06-2.28) | 0.024 |
| **Joint BP-RHR Trajectories** |  |  |  |  |
| Group 1 (Ref) | 1.00 (Reference) | — | 1.00 (Reference) | — |
| Group 2 | 2.15 (1.18-3.92) | 0.012 | 2.29 (1.28-4.09) | 0.007 |
| Group 3 | 1.78 (1.04-3.05) | 0.035 | 1.92 (1.12-3.28) | 0.018 |
| Group 4 | 2.58 (1.12-5.95) | 0.026 | 2.86 (1.26-6.51) | 0.013 |
| Abbreviations: SBP, systolic blood pressure; DBP, diastolic blood pressure; RHR, resting heart rate; BP, blood pressure; IPTW, inverse probability of treatment weighting; HR, hazard ratio; CI, confidence interval; Ref, reference group; NACE, net adverse clinical events. | | | | |
| Sensitivity Analysis 1: The IPTW-adjusted Cox model was additionally adjusted for baseline single-measurement SBP, DBP, and RHR as continuous variables, based on the predefined confounder weighting strategy of the primary analysis. | | | | |
| Sensitivity Analysis 2: The IPTW-adjusted Cox model was constructed after excluding patients with extreme baseline SBP values, with the same confounder balance strategy as the primary analysis. | | | | |

**Supplementary Figure 1.** Individual observed versus predicted mean longitudinal trajectories for the three latent classes of diastolic blood pressure. Notes: This figure illustrates the goodness-of-fit for the identified trajectory model over the 6-month follow-up period. Within each panel (class 1, class 2, and class 3), the bold solid lines represent the model-predicted mean trajectories for that specific class. The lighter, transparent lines represent the actual observed longitudinal measurements of individual patients assigned to the respective class based on their highest posterior probability.

**Supplementary Figure 2.** Individual observed versus predicted mean longitudinal trajectories for the three latent classes of systolic blood pressure. Notes: This figure illustrates the goodness-of-fit for the identified trajectory model over the 6-month follow-up period. Within each panel (class 1, class 2, and class 3), the bold solid lines represent the model-predicted mean trajectories for that specific class. The lighter, transparent lines represent the actual observed longitudinal measurements of individual patients assigned to the respective class based on their highest posterior probability.

**Supplementary Figure 3.** Individual observed versus predicted mean longitudinal trajectories for the two latent classes of resting heart rate.

Notes: This figure illustrates the goodness-of-fit for the identified trajectory model over the 6-month follow-up period. Within each panel (class 1 and class 2), the bold solid lines represent the model-predicted mean trajectories for that specific class. The lighter, transparent lines represent the actual observed longitudinal measurements of individual patients assigned to the respective class based on their highest posterior probability.
